# A Novel Microfluidics Droplet-Based Interdigitated Ring-Shaped Electrode Sensor for Lab-on-a-Chip Applications

**DOI:** 10.3390/mi15060672

**Published:** 2024-05-22

**Authors:** Salomão Moraes da Silva Junior, Luiz Eduardo Bento Ribeiro, Fabiano Fruett, Johan Stiens, Jacobus Willibrordus Swart, Stanislav Moshkalev

**Affiliations:** 1 Electronics & Informatics, Vrije Universiteit of Brussel, 1050 Brussels, Belgium; 2 Center for Semiconductor Components and Nanotechnologies, State University of Campinas, Campinas 13083-852, Brazil; stanisla@unicamp.br; 3 School of Electrical and Computer Engineering, State University of Campinas, Campinas 13083-852, Braziljacobus@unicamp.br (J.W.S.); 4 BioSense Institute, University of Novi Sad, 21000 Novi Sad, Serbia

**Keywords:** microfluidics device, droplet-based microfluidics, lab-on-a-chip sensor, interdigitated electrode, spectroscopic sensing, real-time, microfabrication and soft lithography

## Abstract

This paper presents a comprehensive study focusing on the detection and characterization of droplets with volumes in the nanoliter range. Leveraging the precise control of minute liquid volumes, we introduced a novel spectroscopic on-chip microsensor equipped with integrated microfluidic channels for droplet generation, characterization, and sensing simultaneously. The microsensor, designed with interdigitated ring-shaped electrodes (IRSE) and seamlessly integrated with microfluidic channels, offers enhanced capacitance and impedance signal amplitudes, reproducibility, and reliability in droplet analysis. We were able to make analyses of droplet length in the range of 1.0–6.0 mm, velocity of 0.66–2.51 mm/s, and volume of 1.07 nL–113.46 nL. Experimental results demonstrated that the microsensor’s performance is great in terms of droplet size, velocity, and length, with a significant signal amplitude of capacitance and impedance and real-time detection capabilities, thereby highlighting its potential for facilitating microcapsule reactions and enabling on-site real-time detection for chemical and biosensor analyses on-chip. This droplet-based microfluidics platform has great potential to be directly employed to promote advances in biomedical research, pharmaceuticals, drug discovery, food engineering, flow chemistry, and cosmetics.

## 1. Introduction

Microfluidics has received increasing attention in a broad spectrum of research fields, from fluid physics to biomedicine, due to its capability to handle very small amounts of reactants with a high degree of precision, reliability, reproducibility, and accuracy [1,2,3,4,5]. Microfluidics devices are commonly called lab-on-a-chip (LoC), where microchannels can incorporate multiple unit operations [6,7,8] that, to some extent, replace benchtop laboratory equipment, performing different tasks such as mixing [9], separation, heating, and detection. The benefits of microfluidics technology are based on the small volume of liquid samples [10], which enables faster chemical reactions [11,12] due to the acceleration of mass and heat transfer at the microscale [13,14] and integrated micro actuators [15,16,17]. In the last decades, a microfluidics droplet-based approach rapidly evolved [14,18,19,20,21], largely employed for biomedical applications [16,22,23], especially leading to studies with cells and antibody development [20,24,25], where microfluidics devices have enabled the creation of new tools and protocols [16,26,27,28], for example, for single-cell encapsulation, co-encapsulation, cell-sorting, droplet recovery/extraction (de-oiling), and pico-injection [14,22,24]. The precise control and detection of droplet generation and size are indispensable in numerous microfluidic applications [29,30,31,32], particularly in the field of antibody and drug development [7], including manipulation and delivery in these processes, many of which entail the manipulation of cells or beads inside microchannels for the precise generation and monitoring of microdroplets [8], for which it is necessary to integrate sensing and microfluidics channels [33]. Multiple microfluidics applications have emerged in combination with gas sensors, for example, for monitoring volatile organic compounds (VOCs) in vehicle cabins, a practical and economical approach using microfluidic gas detectors and 3D-printed channels [34,35]. There are important new considerations in microchannel projects regarding geometry, with particular emphasis on the selectivity demonstrated by serpentine channels through simulations using COMSOL Multiphysics [36]. Furthermore, a comprehensive overview of the evolution of microfluidic gas sensors, organizing them according to the target analytes and evaluating key metrics such as specificity, sensitivity, costs, and prospective applications, has been published recently [36].

Optical detection [9], impedance detection [10], and capacitive detection [11] emerge as the predominant sensing techniques utilized for droplet size control and detection. However, it is important to mention that optical-based droplet detection often demands a considerably intricate setup external to the device, involving the introduction of laser light and the subsequent detection of scattered light using optical elements positioned within a microfluidic channel or chamber, as evidenced by studies [12,13]. Optics-based interrogation dependence on external equipment does not allow for the fabrication of portable point of care (POC) [18,37]. Electrochemical sensors can be relatively easy to manufacture and, depending on the specific use case, enable compact circuitry for small and portable devices [10,38,39]. Nonetheless, these sensors have critical limitations; even a very small current passing through the fluid at the open electrode can trigger unwanted reactions such as oxidation, reduction, corrosion, and degradation, leading to inaccurate readings [39,40]. As a result, by affecting sensor longevity and reliability over time, they may not be ideal for long-term monitoring applications. Additionally, they may require more frequent replacement and calibration due to batch-to-batch variation [39]. On the other hand, insulated electrodes enable label-free [41,42] and contactless detection, avoiding degradation on the electrode surface [38,43], as the fluid solutions, in their continuous and dispersive phases, pass over a passivation layer. In this sense, oil droplets in an aqueous solution change the global capacitance and impedance proportionally to the volume of liquids. The sensitivity of the sensor response is directly proportional to the electrode surface/wetted area [43], as the electrode’s geometry gets covered by the droplet composition [38,43]. It is important to note that the fabrication process and performance of planar interdigitated electrodes may vary depending on the specific application and requirements. In order to optimize the fabrication process [40] and enhance the performance of planar interdigitated electrodes, it is crucial to consider the specific application and requirements of the device, as well as the materials and techniques used in the fabrication process [10,38,39,44].

In this study, we introduced a novel design for electrodes in microfluidics sensors called the interdigitated ring-shaped electrode (IRSE), which is fully integrated into a low-cost, droplet-based microfluidics platform. The size of the droplet is found by analyzing (using a LABVIEW 2013-based custom program) individual frames of videos shot from a video camera coupled with a microscope and by monitoring and plotting variations in volumetric flow rate in droplet formation dynamics. As a result, we can identify the various droplet parameters that are correlated with the signal trend and frame analysis videos. We demonstrated precise control and characterization of microdroplets, providing basic droplet parameters such as size, volume, velocity, and shape. We presented a fully integrated microfluidics system capable of detecting oil/water and water/oil emulsions, enabling the simultaneous acquisition of impedance and capacitance spectroscopic data synchronized with video recording. The structure of the IRSE was designed to optimize the capacitance/impedance per unit area ratio, resulting in higher sensitivity, reproducibility, and reliability within a delimited wetted sensing region. Through the integration of the IRSE design into the droplet-based microfluidic architecture, we enhanced the capability for precise characterization of microdroplets for lab-on-a-chip applications.

## 2. Experimental

### 2.1. Microfluidic Channel Fabrication and Sensor Integration

**Figure 1:**
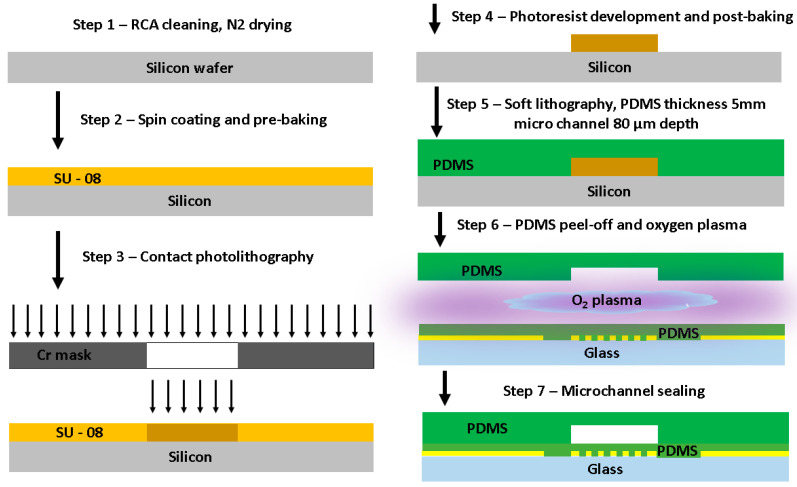
Microchannel fabrication and sensor integration. Soft lithography technique, SU-8 master mold replica, microchannel sealing with oxygen plasma.

A microfluidic channel was fabricated using the soft lithography technique, as reported in our previous works [11,33]. The master mold for the microfluidic channel was fabricated using conventional contact photolithography. The microchannel design pattern was transferred to a silicon wafer coated with SU-8 100 photoresist. A few microchannel replicas were fabricated using a monomer material (Sylgard 184, Midland, MI, USA) deposited onto the master mold. The monomer material used was polydimethylsiloxane (PDMS). A layer of PDMS was deposited in the SU-8 mold. PDMS comprises two parts: a curing agent and a prepolymer base. They were mixed by stirring at a 1:10 weight ratio. The mixture was poured onto a replication master and degassed in a desiccator at 5.3–6.7 Pa for 120 min to eliminate all trapped air bubbles in the mixture. Figure 1 shows the simplified version of the fabricated device. For more details on microfabrication steps, see the Supplementary Material. Masks were fabricated for lithography and thin film deposition via sputtering and lift-off.

The master mold was fully covered with an uncured PDMS mixture; the next step was to get the polymer cured. The curing process was carried out for 60 min on a hot plate at 100 °C and was followed by cooling down to room temperature to peel the PDMS layer from the mold. Subsequently, the microchannel sealing process was carried out with oxygen plasma by oxidizing PDMS and microscope slide surfaces through RF O_2_ plasma (plasma cleaner, PLAB SE80) for 2 min. Finally, the PDMS microchannel layer and the glass substrate containing the IRSE were manually aligned under the microscope and attached together to allow the complete bonding process between the two surfaces to take place for 2 h, finalizing the device fabrication at room temperature, as shown in Figure 1 (step 7). The channel dimensions were: 200 µm width, 80 µm height, and 1 cm length. The criteria used to define channel dimensions are based on our previous work [11,33], aiming to keep the Reynolds number low enough to maintain the laminar flow regime, which is more commonly used for droplet formation. To define the IRSE design, we got insights from previously reported works [45,46] based on the use of curved and spiral electrodes. The electrode design consists of 29 pairs with a 500 nm metal layer thickness. Electrodes, manufactured at a low cost on a glass substrate, feature a 10 µm width and a 10 µm gap. The outer radius of the electrode is 600 µm, resulting in a total occupied area of 1.13 mm^2^.

More details on the fabrication process are presented in the Supplementary Material.

### 2.2. Experimental Setup

**Figure 2:**
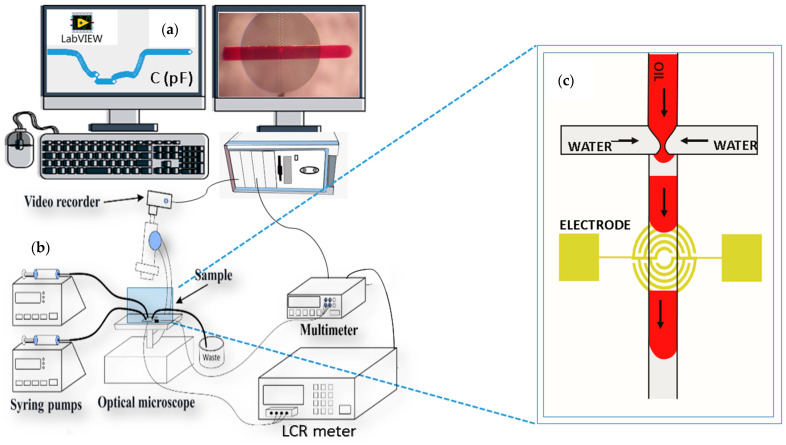
Microfluidics experimental setup used to acquire the capacitive/impedance response. (**a**) LabView user interface and image acquisition. (**b**) Platform with syringe pumps, LCR meter, multimeter, and microscope. (**c**) Microfluidics droplet generator with integrated IRSE sensor.

Once the microfluidics chips were fabricated, we examined them under a microscope and performed leakage tests. Leakage tests were done with an experimental setup, as shown in Figure 2. We began evaluating the bonding quality by checking the channel leakage on a qualitative basis.

This was done by ramping up the volumetric flow rate (VFR) in the inlets with multiple incremental steps until the channel burst or any leakage was observed. The test was performed as follows: with 2 syringe pumps simultaneously connected to each inlet, we started with a very low rate of 60 µL/h, incrementing to the following VFR values: 3000 µL/h and above (6000 µL/h, 12,000 µL/h, and further up to 60,000 µL/h, adding 6000 µL/h each time). We started to notice leakages at a VFR above 3000 µL/hour in the 2 channel inlets after 20 min of usage. The majority of VFR used in this work was far below the burst point. The criteria for choosing the VFR range are to consider laminar flow (low Reynolds number), facilitate droplet formation, and prevent microchannel leakage.

To visually evaluate the droplet formation process, size, and velocity, we attached a CCD camera to the microscope and simultaneously recorded videos. Individual frames of videos were analyzed using the VLC extension and AutoCAD. The signal changes generated on the IRSE sensor were continuously received via user-friendly software (using a LabVIEW 2013-based custom program). In parallel, we connected the LCR meter (Hewlett-Packard, 4284A) to the IRSE to monitor real-time signal changes in terms of capacitance and impedance over the IRSE sensor. The full setup was connected to a computer with software developed in LabView^®^ 2013 that performs the data acquisition and displays it. The results and analyses were performed on the post-processed images and videos synchronized with the data signal recorded from the sensor over time. Since fluid permittivity varies with temperature, the device temperature was monitored to ensure it was kept near room temperature. We used a benchtop multimeter (Agilent, 34401A) connected to a thermocouple (PT100) as a reference temperature sensor placed in contact with the microfluidic chips, as shown in Figure 2.

## 3. Results and Discussion

### Droplet Formation and Detection Mechanisms

Multiphase microflows are characterized by the ratio of viscous to surface forces, the capillary number (***Ca***), and the Webber number (***We***) by the ratio of fluid viscosities, as described in Equations (1) and (2) [12,47]:

(1) $$Ca=\frac{\mu U_{d}}{\sigma }\frac{\mu }{\mu_{d}}$$

and

(2) $$We=\frac{\rho U_{d}^{2}d_{h}}{\sigma }$$

where ***µ*** and ***µ_d_*** are the fluid viscosities of the continuous and the dispersed phases, respectively.

***U_d_*** is the velocity, **σ** is the density, ***ρ*** is the density, and ***d_h_*** is the hydraulic diameter of the channel.

**Figure 3:**
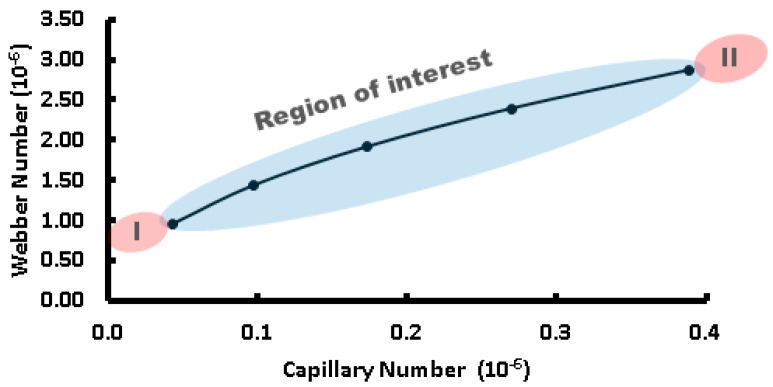
Regime flow relation of Webber and capillary numbers in droplet formation dependency. Highlighted areas: in blue, the region of interest for droplet formation; in red, the regions (I) and (II) are very unstable, forming droplets with large size variations.

The ***Ca*** number represents the balance between viscous forces and surface tension forces, whereas the ***We*** number indicates the relative importance of inertial forces compared to surface tension forces. The interaction between the capillary and Weber numbers significantly influences the process of droplet formation in microfluidic systems, as shown in Figure 3. At lower ***Ca*** values, where viscous forces are dominant over surface tension forces, droplet formation occurs more slowly and tends to be more spherical in terms of shape due to the prevalence of surface tension effects [47]. As ***Ca*** increases, surface tension’s influence decreases, resulting in faster droplet formation and non-spherical shapes as inertial forces become more significant. Similarly, the Weber number has a significant influence on the droplet formation dynamics.

At low ***We*** values, where surface tension dominates over inertial forces, small spherical droplets can be formed primarily due to surface tension effects. However, at higher ***We*** numbers, inertial force becomes greater, leading to quicker droplet generation along with the potential for satellite or irregular-shaped droplet formations. To determine the type of regime flow, the Reynolds number is important; it can be calculated by the ratio of ***We*** and ***Ca***, as follows: ***Re*** = ***We/Ca***. Our droplet formation regime flow was defined by a fixed volumetric flow for the dispersive phase (in our case HFE 7500 oil from 3 M) of 8 µL/h, 21 µL/h, and 34 µL/h and for the continuous phase (DI water) of 1 µL/h, so the Reynolds number could be extracted.

The droplet formation occurs in a very laminar flow regime, with ***Re*** ranging from 0.0452 to 0.1357, as shown in Figure 3. Understanding these dynamics is crucial for optimizing processes related to microdroplet generation, stability, control, size, and shape across droplet-based microfluidics application platforms. In a steady state, after priming the channel with the dispersive phase and injecting the dispersive phase with small increments of volumetric flow rate, very precise control of droplet formation is required.

**Figure 4:**
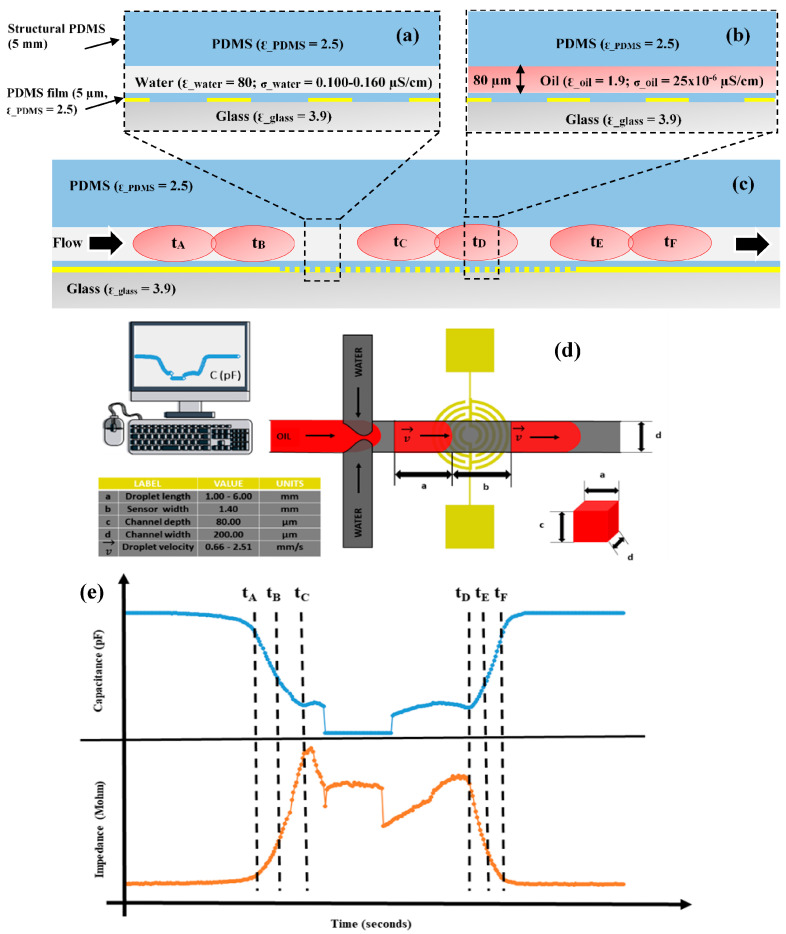
Cross section of IRSE sensor structure and droplet response. (**a**) Microfluidics channel filled with the continuous phase and (**b**) filled with the dispersive phase (microchannel dimensions, dielectric constants of materials, and conductivities of liquids are shown). (**c**) Flow direction and droplet response with droplet movement over time. (**d**) Droplet generation design in a double-junction channel: spontaneous droplet formation response. (**e**) Real-time droplet response in capacitance and impedance, as indicated time from ***t_A_*** to ***t_F_***, droplet dynamics progressing over the sensor.

**Figure 5:**
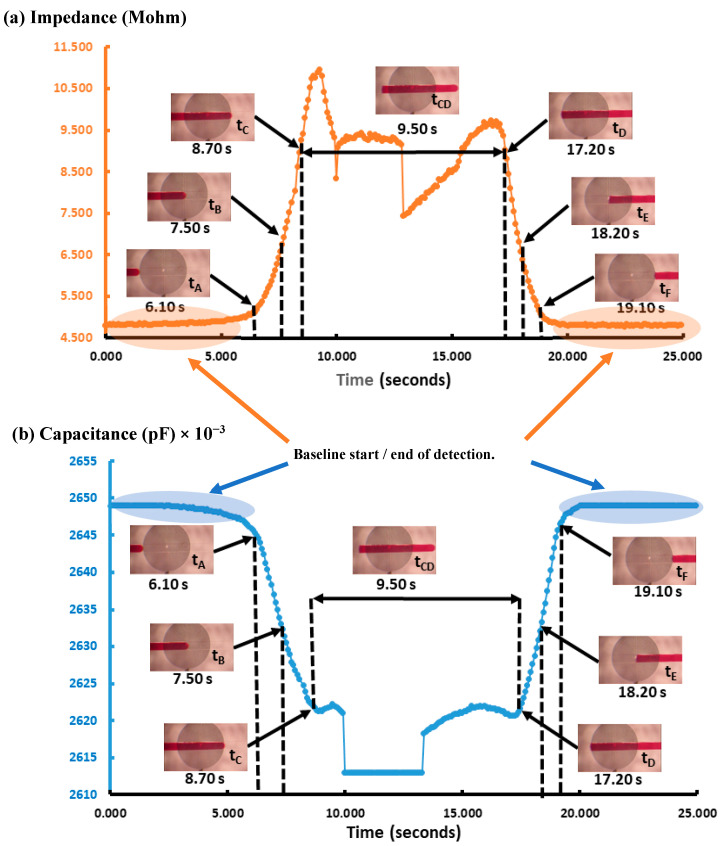
The IRSE sensor’s response to droplets over time. (**a**) Impedance and (**b**) capacitance. Real-time detection droplet dynamics, as indicated at time: ***t_A_***, ***t_B_***, ***t_C_***, ***t_CD_***, ***t_D_***, ***t_E_***, and ***t_F_***. Start of detection: the continuous-phase signal drops slightly before ***t_A_***; at ***t_A_***, the droplet is approaching the edge of the sensor; at ***t_B_***, 50% of the sensor is covered by the droplet; at ***t_C_***, ***t_CD_***, and ***t_D_***, the sensor is fully covered by the droplet; at ***t_E_***, 50% of the sensor is uncovered by the droplet; at ***t_F_***, the droplet is exiting the sensor edge. End of detection: after ***t_E_***, the sensor response returns to the initial continuous phase level.

The ***We*** and ***Ca*** numbers play a crucial role in size variation and stability over time. The IRSE sensor was combined with a double T-junction microchannel to assess the droplet formation, size, and velocity, as shown in Figure 4 and Figure 5. The spontaneous generation of droplets was managed using two syringe pumps (NE100, New Era Pump Systems, Wantagh, NY, USA). As previously mentioned, the continuous phase consisted of ultra-pure DI water with a high resistivity value (18.2 MΩ·cm), while the dispersed phase comprised fluorinated oil HFE7500. To improve droplet formation visibility, the dispersed phase was colored red to enhance contrast and enable data synchronization between the camera and the sensor. To determine the steady baseline, first we recorded the signal of the continuous phase, followed by the trend line of the dispersive phase, and then the continuous phase again, as shown in Figure 4. The sensor operation principle is electrical, employing a capacitive mode to detect variations in dielectric materials and liquids within the IRSE area. In the impedance mode, sensors measure the overall liquid’s conductance over time. In the capacitive mode, our sensors detect changes in electrical capacitance caused by the presence of different dielectric materials or liquids on the sensor’s surface, translating this into a signal indicating specific substances, as we demonstrated for water and oil emulsions. Impedance sensors assess the overall liquid’s conductance over time, providing comprehensive information on its properties and behavior. By integrating an electrode sensor within microfluidics channels, our system enables more precise analysis of liquid properties with enhanced sensitivity and accuracy, facilitating the characterization of diverse substances through real-time monitoring and analysis. Previous works reported the usage of interdigitated electrodes (IDEs) and serpentine electrodes [45], where the serpentine electrode showed better performance and results. In another study, different IDEs were compared, including classical IDE, serpentine, and spiral electrodes [46].

Overall, curved electrodes demonstrated better performance in capacitance and sensitivity/area. Based on this, we engineered a novel design featuring an expanded sensitivity area and higher capacitance in comparison with traditional IDEs. Here, we designed a spiral interdigitated electrode that gives great benefits in terms of the footprint size of the electrode, sensitivity/area, and capacitance due to the increased confined electrial field.

This integrated approach enhances efficiency and reliability in liquid sensing applications by promptly identifying changes. In summary, the integration of capacitive and impedance-sensing technologies enables more accurate and sensitive analysis of liquid properties, leading to improved understanding and characterization of different substances in real time, thereby enhancing efficiency and reliability in liquid sensing applications.

**Figure 6:**
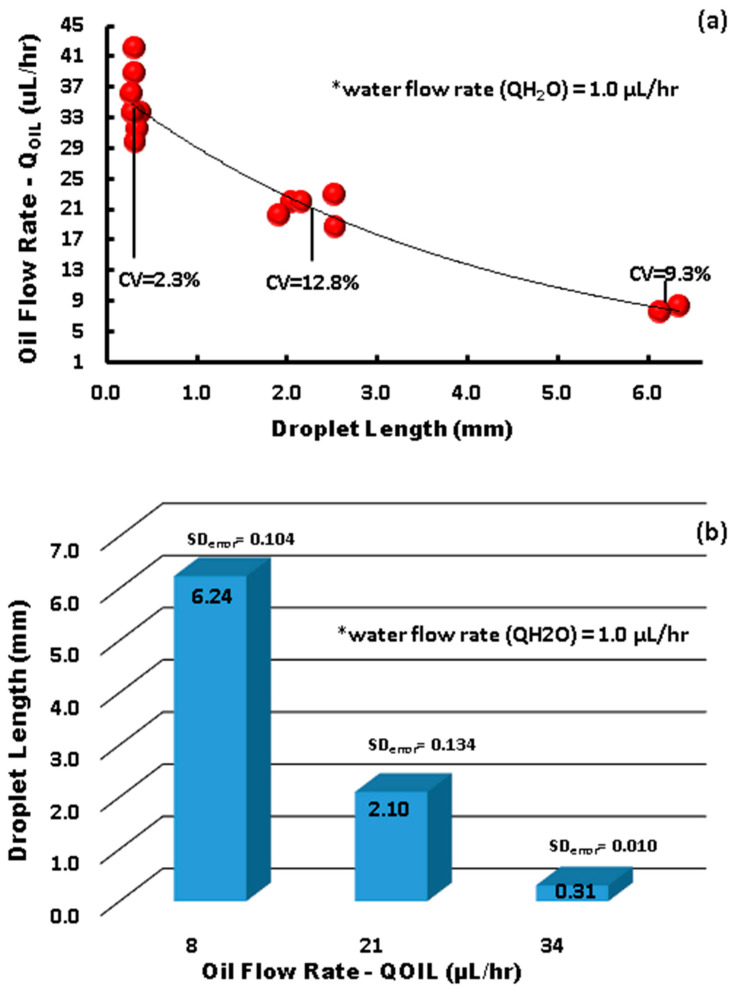
Microchannel droplet formation (* total volumetric flow rate fixed). (**a**) Three data points analyzed droplet length dependency on flow rate, with a calculated coefficient of variance (CV%). (**b**) Droplet size distribution and size variation, with calculated standard error.

As can be observed in Figure 5, the impedance signal generated by the droplets on the sensor has a peak at 10.60 MΩ and a baseline around 4.60 MΩ, leading to a very high signal amplitude of 6 MΩ. On the other hand, in terms of capacitance, the peak was at 2.650 × 10^−2^ pF and the baseline was around 2.615 × 10^−2^ pF, leading to a signal amplitude of 35 × 10^−2^ pF, with sensitivity being 12 times higher than that reported by Ernst et al. [41]. It is worth mentioning that curved and spiral electrodes have performances 1.5 times higher in comparison with IDE, as reported in [45,46]. The sensitivity of our IRSE sensor agrees with previously reported work [46]. They demonstrated it with a pressure sensor and humidity application. The correlation between droplet length and VFR is shown in Figure 6. In Figure 6a, we have a plot of droplet length as a function of VFR and length coefficient variation for each case. We achieved CV 2.3–12.8% droplet length variation for a fixed and constant VFR of continuous phase (1 µL/h) and varying the dispersive phase from 7 to 41 µL/h. Figure 6b shows three populations of droplets formed, respectively, for three given VRFs of 8 µL/h, 21 µL/h, and 34 µL/h and again water VFR fix at 1 µL/h. The plot shows the three lengths of droplets formed: 6.24 mm, 2.1 mm, and 0.301 mm and their respective standard errors.

A unique pair of volumetric flow rates set on the syringe pumps determined the droplet size and shape. There are three main droplet formation regimes: (I) a dripping regime, which produces small spherical droplets; (II) a regime of interest with medium-sized droplet formation; (III) a squeezing regime, which produces long plug-like droplets. We used the squeezing regime to evaluate the sensor response due to limitations in the real-time response of the electrode sensor and the wetted area required to avoid false readings of the droplet length, which must be equal to or bigger than the electrode length. In Figure 6a,b, we delimited the regime flow into three data points, generating as a result three populations of droplet size: small, medium, and large.

**Figure 7:**
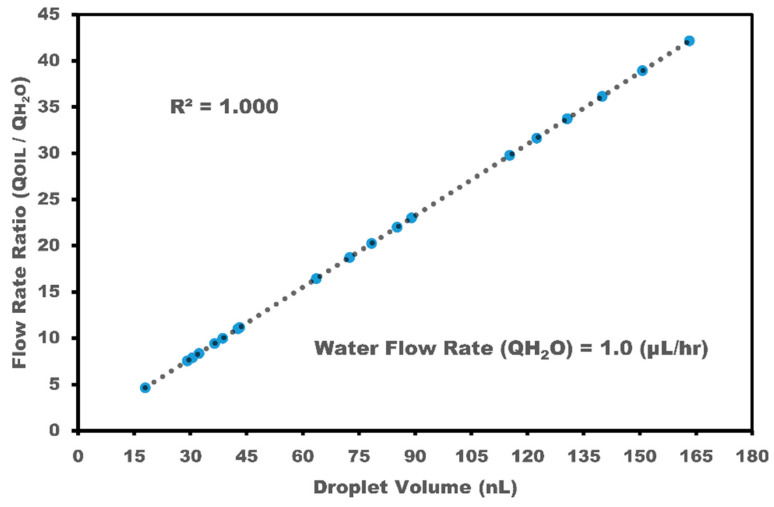
Droplet volume depends on the volumetric flow rate, with a calculated standard error smaller than 0.134.

In fact, the small group of droplets is not detectable because the sensor averages the signal with no clear response. The impedance and capacitance change dynamically, in accordance with the droplet passage over the IRSE sensor. To relate the output signal to the flow rate parameters, images of droplet formation were captured with a camera coupled to a microscope, and the sensor readout was validated with images and video acquired from droplet generation. These enabled measurements of droplet size, length, and volume, validating the sensor readout through video technique extraction. Figure 7 shows the data extracted directly from videos synchronized with the IRSE sensor response at the same time. We extracted the timestamp from acquired images and videos and then multiplied it by sensor length. With this approach, we were able to estimate the droplet’s velocity, length, and volume. For more details and plots comparing video extract versus stack images in VLC software (Version 3.0.20) and AutoCAD 2022, see the Supplementary Material.

Figure 7 shows the droplet volume dependency with volumetric flow rate. Note that it is possible to reduce the droplet size to less than the distance occupied by the sensor by increasing the flow of aqueous colorant relative to the oil flow. In this case, the impedance does not reach its peak value; it gets in the middle of the amplitude. Further, if two or more droplets enter the sensor field, the measured value is relative to the average impedance over the IRSE. Each droplet of aqueous colorant represents an increase in IRSE impedance because oil has higher resistance than water and a higher dielectric constant, leading to higher capacitance. Electrode geometry is a critical parameter for electrode sensitivity. The analysis of impedance and capacitance biosensors in interdigitated electrodes (IDEs) hinges significantly on electrode geometry. Fine-tuning the interdigitated electrode’s width, spacing, and active area allows for precise impedance and capacitance output adjustments. As demonstrated by H. Pandya and W. Zhang et al., employing a spacing of 10 μm as opposed to 30 μm yields a remarkable ~69% increase in the disparity of impedance between benign and cancerous breast tissue samples [48,49]. These results inspired us to define our electrode geometry with an electrode width of 10 um and spacing of 10 um. T. Dong and W. Zhang et al. demonstrated a correlation between the initial capacitance and the number of active finger pairs. As the number of active finger pairs increases, the initial capacitance also increases, indicating a higher density of electrical field lines over the sensing area [49,50]. Based on these findings, we decided to design our electrode with a small footprint area (1.13 mm^2^) sufficient to integrate with microfluidic channels and sensitivity sufficient to detect biphasic droplets.

When capacitance exceeds 2.650 × 10^−2^ pF, the pulse length determines the time that the droplet was over the IRSE sensor working in capacitor mode. On the other hand, the base value is kept when capacitance is about 2.610 × 10^−2^ pF, which determines the period in which the continuous-phase fluid is over the IRSE. Considering the flow rate and the microchannel cross section, the droplet absolute velocity can be calculated by the droplet-traveled distance in the microchannel volume that can be taken from the cross-sectional area of the microchannel and droplet length. We observed that the flow rate imposed a proportional change in the velocity and volume of the droplets. In addition, it relates to variables of interest such as signal changes; pulse length when impedance is over 10.50 MΩ determines the time that the droplet was over the IRSE sensor working in impedance mode, while the base value is kept when impedance is about 4.50 MΩ, determining the period in which the continuous-phase fluid is over IRSE. Finally, we have presented a fully integrated microfluidics droplet-based system applied for the generation and detection of nanovolume droplets with both impedance and capacitance responses.

It is essential to have additional components in lab-on-a-chip applications, combining microelectrode sensors and microchannels, as we demonstrated in this work with a novel electrode design. These sensors, fabricated in a cleanroom environment using precise microfabrication techniques, comprise closely positioned concentric ring-shaped electrodes that allow for the efficient handling and detection of analytes, particularly charged species such as ions or biomolecules. In addition, they are sterile and suitable for biomedical applications. Because of their very small size and compatibility with microfabrication methods, they can be integrated into microfluidic devices for the accurate on-chip detection and analysis of analytes. In this case, we have demonstrated droplet formation and detection simultaneously. The precise control provided by microfabrication techniques ensures the creation of strong and confined electric fields in a small area between the concentric rings with a 10 µm gap, thus improving sensitivity and enabling the dynamic manipulation of analytes within the microfluidic environment. As a result, IRSE sensors are likely to play a crucial role in advancing lab-on-a-chip technology by offering high sensitivity and specificity for various applications, from biomedical diagnostics to environmental monitoring, highlighting their significance in the analytical sciences.

## 4. Conclusions

In this study, we presented the results of the development of a novel IRSE (interdigitated ring-shaped electrode) microfluidic device capable of generating and simultaneously characterizing microdroplets with nanoliter volumes. By integrating electrical (capacitive and impedance) and optical (droplet shape and size) characterizations, the system allows for precise, fully controllable, and reproducible droplet generation. The integration of various on-line characterization techniques, as well as enhanced sensitivity of electrical measurements due to a special design of electrodes, is a distinctive feature of the present design, resulting in greatly enhanced functionality of the device.

We employed a double T-junction-shaped microchannel within a multiphase microfluidic system to generate droplets of oil in water (o/w). The dispersive phase consisted of oil mixed with a red dye colorant, while DI water was used as a continuous phase. The real-time synchronization of sensor data with video recordings facilitated the analysis and validation of all acquired data. Our findings consistently revealed impedance and capacitance variations corresponding to biphasic fluid flowing over the sensor, with signal changes in terms of capacitance 35 × 10^−2^ pF and impedance 6.0 MΩ. Using this new sensor design, we were able to make analyses of droplet length in the range of 1.0–6.0 mm, velocity of 0.66–2.51 mm/s, and droplet volume of 1.07 nL–113.46 nL. We have demonstrated a high-sensitivity IRSE sensor, with sensitivity being 12 times higher than that reported by Ernst et al. [41], also in agreement with previously reported work [46].

The utilization of integrated real-time capacitance and impedance measurements holds significant promise in microfluidic devices, particularly for the precise control of desired droplet sizes in biphasic mixtures. This novel sensor integration with microfluidic channels has great potential for numerous practical applications, including drug delivery and food encapsulation. The microdroplet generator results in a very linear correlation between droplet length and volumetric flow rate, leading to droplet length as a function of VFR with a small coefficient of variation, ranging from CV 2.35–12.8%. Furthermore, in the dripping regime, the IRSE sensor allows for droplet velocity and volume estimation, enhancing its utility in microfluidic systems. The integration of real-time capacitance and impedance measurements into microfluidic platforms offers more precise control over droplet formation and flow rates, with implications extending across flow chemistry, biopharmaceuticals, diagnostics, and chemical synthesis. We demonstrated a significant capacitance and impedance response per area, demonstrating that the sensor footprint is small enough to integrate into microfluidic channels and sensitive enough to detect biphasic droplets in real time. Furthermore, the IRSE sensor enables real-time monitoring and analysis of droplet dynamics, providing invaluable insights into droplet behavior within microfluidic contexts.

## Data Availability

The original contributions presented in the study are included in the article, further inquiries can be directed to the corresponding authors.

## Supplementary Materials

The following supporting information can be downloaded at: https://www.mdpi.com/article/10.3390/mi15060672/s1, File S1: droplets; Video S1: droplet formation.
