# Supplementary material for: A Novel Microfluidics Droplet-Based Interdigitated Ring-Shaped Electrode Sensor for Lab-on-a-Chip Applications
Source: Micromachines (Basel). 2024 May 22;15(6):672. doi: 10.3390/mi15060672 (PMC11205656; doi:10.3390/mi15060672)
Supplement: Supplementary file 1 [file micromachines-15-00672-s001.zip › 4431550-supplementary-final.pdf]

Supplementary Material

Salomão Moraes da Silva Junior[1,2,3,4,*], Luiz Eduardo Bento Ribeiro[3], Fabiano Fruett[3], Jacobus Willibrordus Swart[2,3], Stanislav Moshkalev[2,3], Johan Stiens[1]

[1]Electronics & Informatics, Vrije Universiteit of Brussel, Brussels 1050, Belgium

[2]Center for Semiconductor Components and Nanotechnologies, State University of Campinas, Campinas 13083-852, Brazil

[3]School of Electrical and Computer Engineering, State University of Campinas, Campinas 13083-852, Brazil

[4]BioSense Institute, University of Novi Sad, Novi Sad 21000, Serbia

*author to whom correspondence should be addressed.

E-mail: salomaomoraes@yahoo.com.br

Photomask fabrication

Two masks were fabricated, one for the fluidic microchannels and the second for the microelectrode. Masks can be microfabricated using direct laser writing, thin film deposition, and lift-off. The fabrication process started with IRSE and microfluidic channel patterns; the design was made using CAD software (AutoCAD®, Autodesk, 2007). The masks were made of high-resolution glass from Kodak (60 mm side Kodak® 1A).

The fabrication process follows glass substrate cleaning using Standard RCA (Radio Corporation of America). After cleaning and drying with nitrogen gas, the glass was placed on a hot plate at 120ºC for 20 minutes. After that, a very thin layer of chromium (*Cr*), 10 nm, was deposited, and then a thin layer of gold (*Au*), 150 nm, was deposited.

Next, a spin coating technique was used to apply a photoresist layer (AZ 1518, Merck). The pattern was transferred using high-resolution direct laser writing photolithography (Model µPG101, Heidelberg Instruments). The unprotected Cr/Au were chemically etched using a mixture of nitric acid and hydrochloric acid (1:3 ratio) at room temperature after exposure, post-baking, and development. The strong oxidative nature of this mixture, resulting from the combination of nitrosyl chloride and free radicals, dissolved the metal layer, while Aqua regia consumed itself and decomposed, forming nitrous and chlorine gases [1,2]. After that, the substrates were then rinsed with DI water (18.2 MΩ.cm) and dried with nitrogen (N_2).

Microelectrode fabrication

Photolithography of IRSE microelectrode: Using the IRSE electrode mask and conventional broadband photolithography, the patterns were transferred into a glass substrate previously coated with a metal layer using a lift-off technique [3]. This process was conducted with a contact mask aligner (MJB-3 UV300 Karl-Suss, Garching, Germany), after spin-coating the substrate with AZ 1518 (1.8µm thick), @ 4000 rpm for 30 seconds, as shown in Figure S1. Electrode Deposition and Lift-Off: A thin film of titanium-gold-titanium (Ti/Au/Ti) was deposited using an e-beam evaporator (Leybold Univex 300, Cologne, Germany), to improve soldering quality in the electrode terminals. Following deposition, devices underwent a lift-off technique, immersing them in acetone to remove the photoresist layer and excess metal, resulting in the desired IRSE-patterned electrodes on the glass surface. These techniques were used in our previous works [4–6]. The electrode design consists of 29 pairs with a 500 nm metal layer thickness. Electrodes, manufactured at a low cost on a glass substrate, feature a 10µm width and a 10µm gap. The outer radius of the electrode is 600µm, resulting in a total occupied area of 1.13mm².

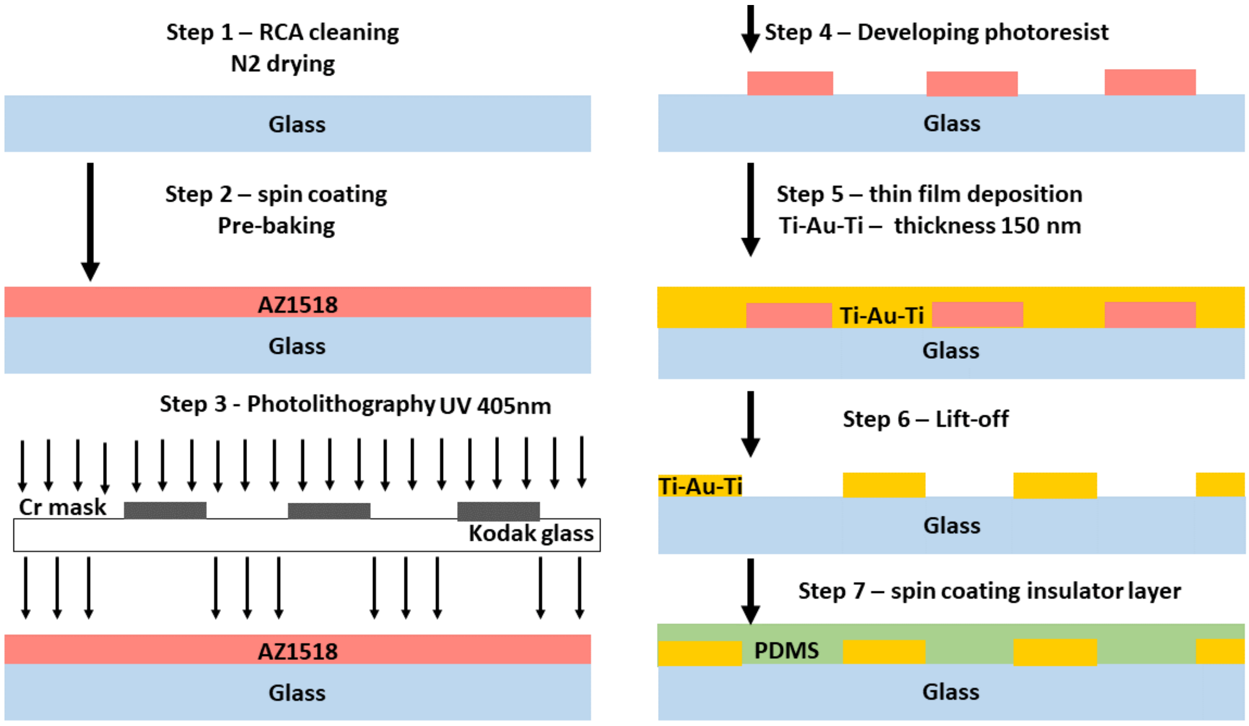

Figure S1. Microfabrication of masks using direct laser writing techniques, thin film deposition, and Ti-Au-Ti lift-off.

Electrode passivation

Passivation with polydimethylsiloxane (PDMS): As a final step, a Silicone Elastomer Kit (Sylgard 184, Dow Corning, Midland, USA) was used as a passivation material. PDMS, known for its low-k value (2.5), serves as an effective insulator, protecting electrodes from degradation. A thin and uniform PDMS layer with a 5µm thickness was deposited using the spin coating technique, as shown in Figure S2. Based on previously reported online information from the Elvesys website, we chose a three-speed rotation to make PDMS spin-coating deposition. For the 8000 rpm rotation, the results were not uniform and consistent; the PDMS film was not evenly distributed on the surface. For 2000 rpm, the insulation layer was too thick, around 35-45 µm, leading to sensor signal response instabilities. Our best coating was at 5000 rpm, resulting in a PDMS film of 5 µm and consistent results.

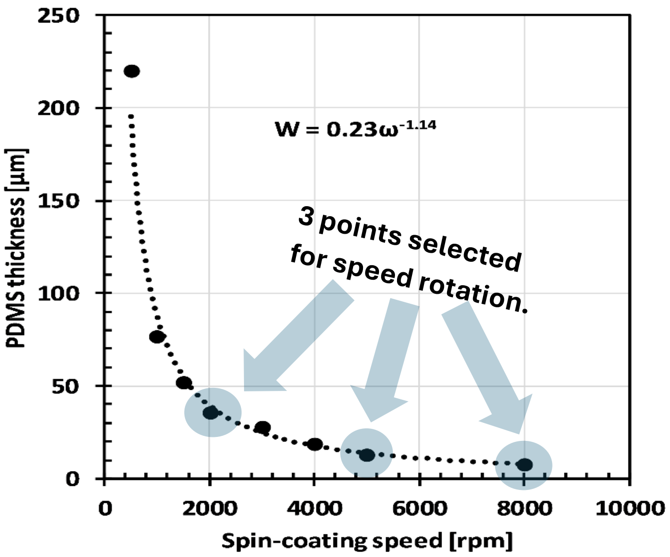

Figure S2. PDMS film thickness in function of speed rotation in [rpm], which ***W*** is the film thickness, and ***w*** is the speed., adapted from Elvesys website.

Droplet analyses

Droplets were analyzed by video time stamp and VLC extension time in milliseconds (ms) and image stack pile analysis in AutoCAD, as shown in Figure S3 (a) and (b), and criteria were used to analyze the head and tail of droplets individually. Figure S4 shows the full analysis, head-to-tail droplet analysis, and comparison between the approaches.

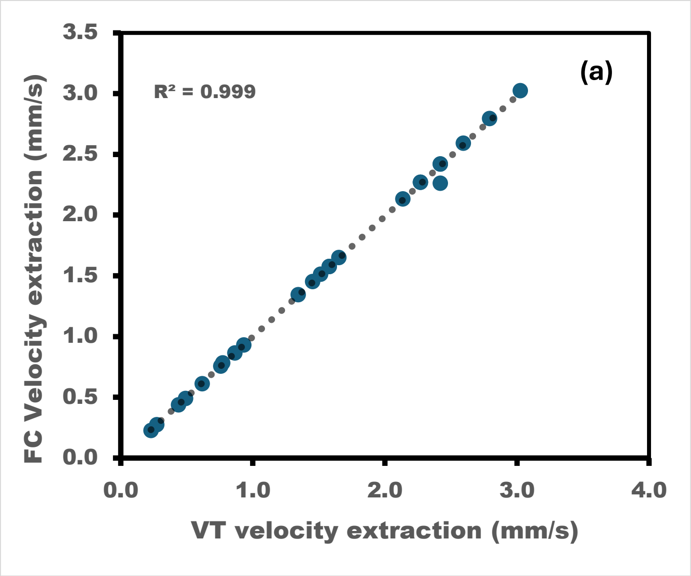

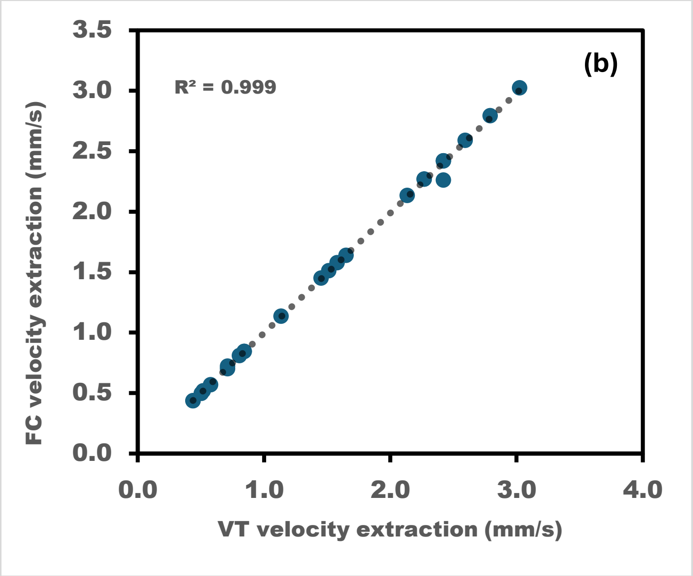

Figure S3. (a) Comparison Techniques Video Frame Counting versus Video Timestamp (HEAD). (b) Comparison Techniques Video Frame Counting versus Video Timestamp (TAIL).

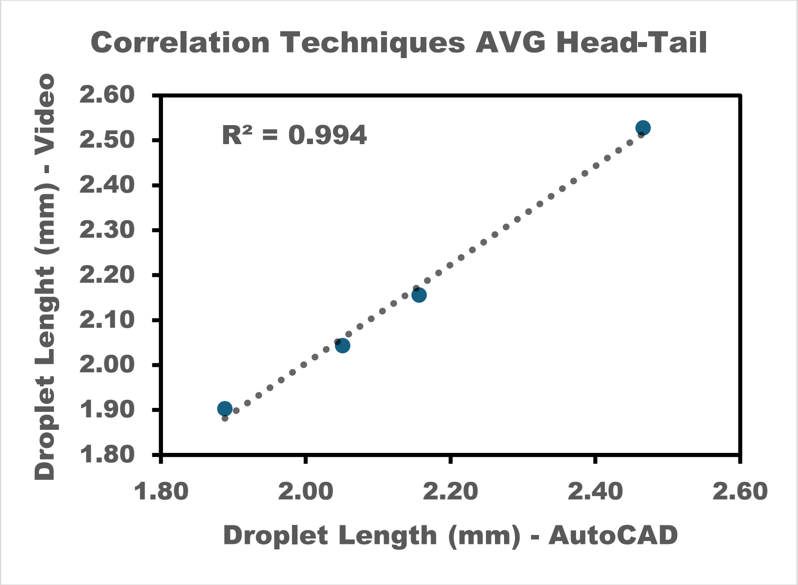

Figure S4. Comparison Techniques Video Frame Counting versus Video Timestamp (HEAD-to-TAIL). Correlation Techniques AVG Head-Tail AutoCAD and VLC extension.

References

1. Xu, Z.; Wang, Y.; Sheng, K.; Rosenthal, R.; Liu, N.; Hua, X.; Zhang, T.; Chen, J.; Song, M.; Lv, Y.; et al. Droplet-Based High-Throughput Single Microbe RNA Sequencing by smRandom-Seq. *Nat Commun* **2023**, *14*, 5130, doi:10.1038/s41467-023-40137-9.
2. Uhlen, M.; Quake, S.R. Sequential Sequencing by Synthesis and the Next-Generation Sequencing Revolution. *Trends in Biotechnology* **2023**, *41*, 1565–1572, doi:10.1016/j.tibtech.2023.06.007.
3. Ribeiro, L.E.B.; de ALCÂNTARA, G.P.; Andrade, C.M.G.; Fruett, F. Analysis of the Planar Electrode Morphology Applied to Zeolite Based Chemical Sensors. **2015**, *193*.
4. Da Silva Junior, S.M.; Stiens, J.; Moshkalev, S.; Swart, J.W.; Matvejev, V.; Zhang, Y.; De Tandt, C. Subterahertz Sensor in Microfluidic Devices for On-Line Determination and Control of Ethanol Concentration. *Journal of Vacuum Science & Technology B, Nanotechnology and Microelectronics: Materials, Processing, Measurement, and Phenomena* **2017**, *35*, 06GA02, doi:10.1116/1.4991891.
5. Flacker, A.; Adamo, C.; Da Silva Junior, S.; Silva, M.; Mederos, M.; Teixeira, R. WET TREATMENT AND THE BEHAVIOR OF ELECTROLESS NI-P DEPOSITION AT 40 °C ON POLISHED ALUMINA. *Quim. Nova* **2023**, doi:10.21577/0100-4042.20230061.
6. Moraes Da Silva Junior, S.; Stiens, J.; Moshkalev, S.; Willibrordus Swart, J.; Lacerda De Orio, R.; Matvejev, V.; Zhang, Y.; Vandermeiren, W.; De Tandt, C. Microfluidic Devices on Glass for Liquid Mixtures Concentration with Coupled Thz Sensor. *JICS* **2018**, *13*, 1–5, doi:10.29292/jics.v13i2.10.
