# Supplementary figures and images for: A Novel Microfluidics Droplet-Based Interdigitated Ring-Shaped Electrode Sensor for Lab-on-a-Chip Applications
Source: Micromachines (Basel). 2024 May 22;15(6):672. doi: 10.3390/mi15060672 (PMC11205656; doi:10.3390/mi15060672)

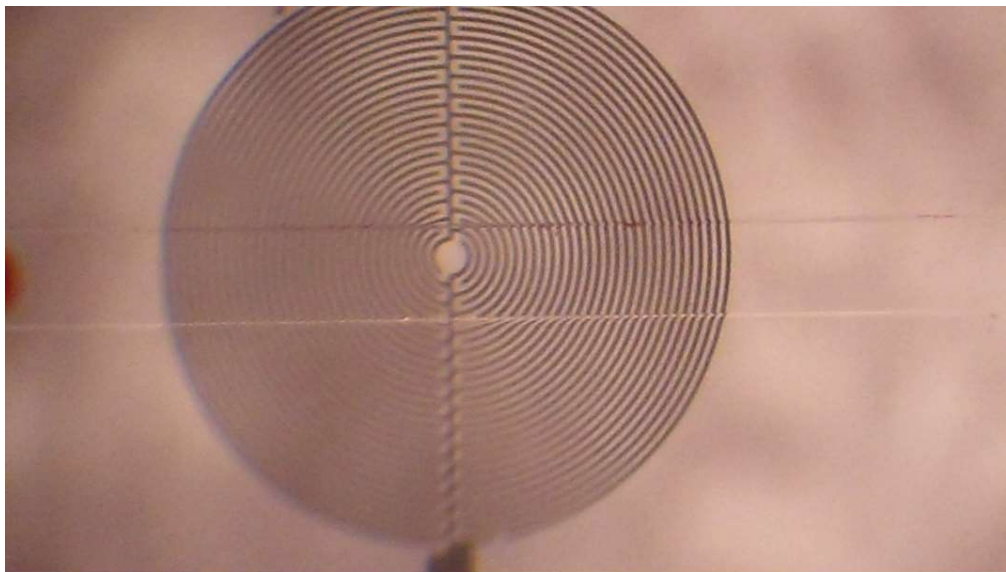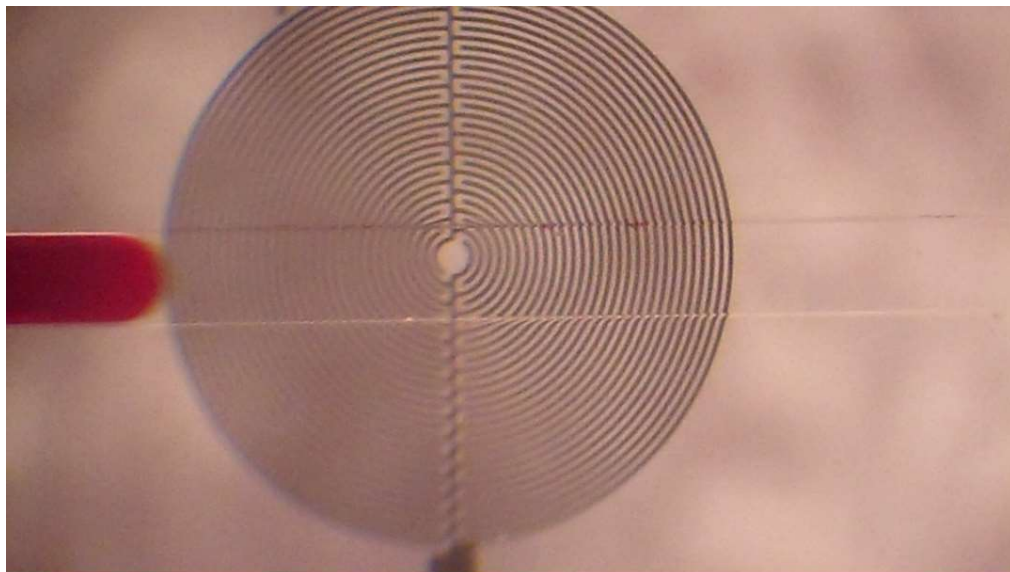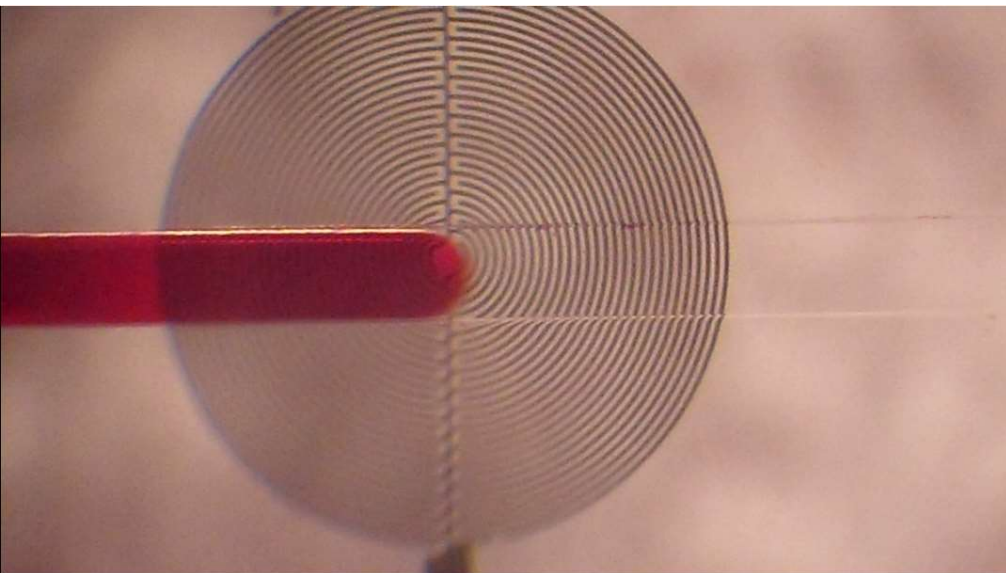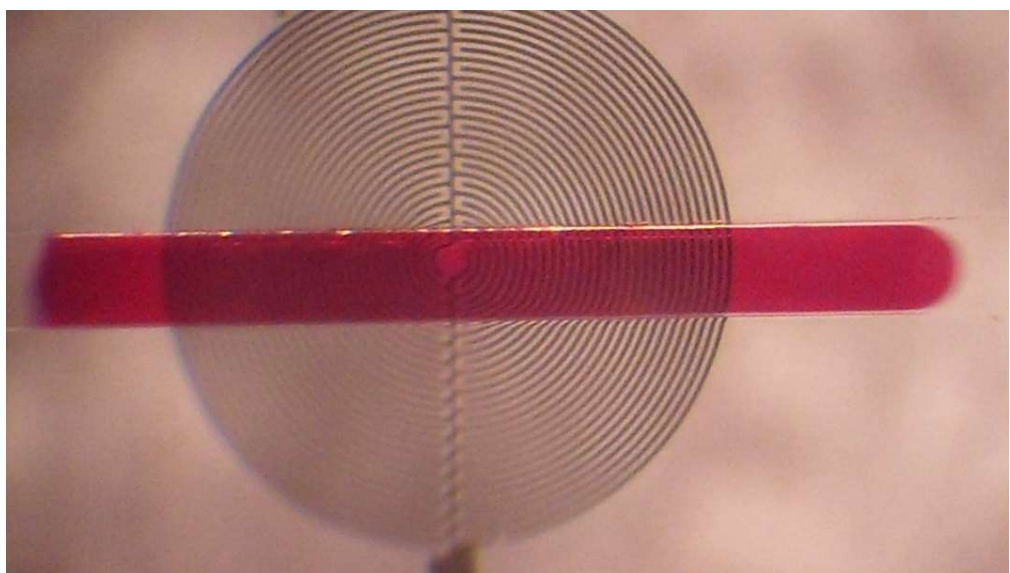

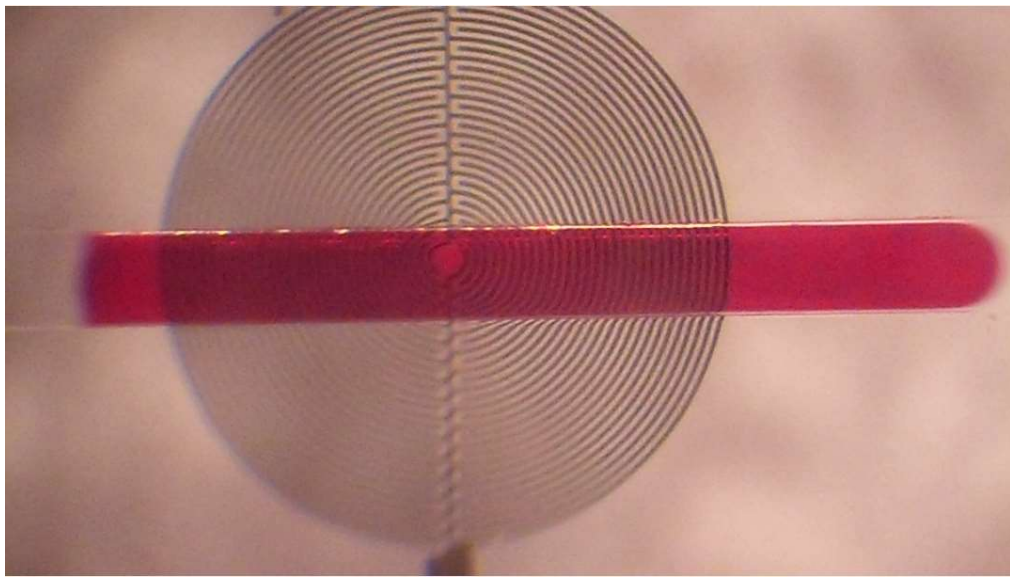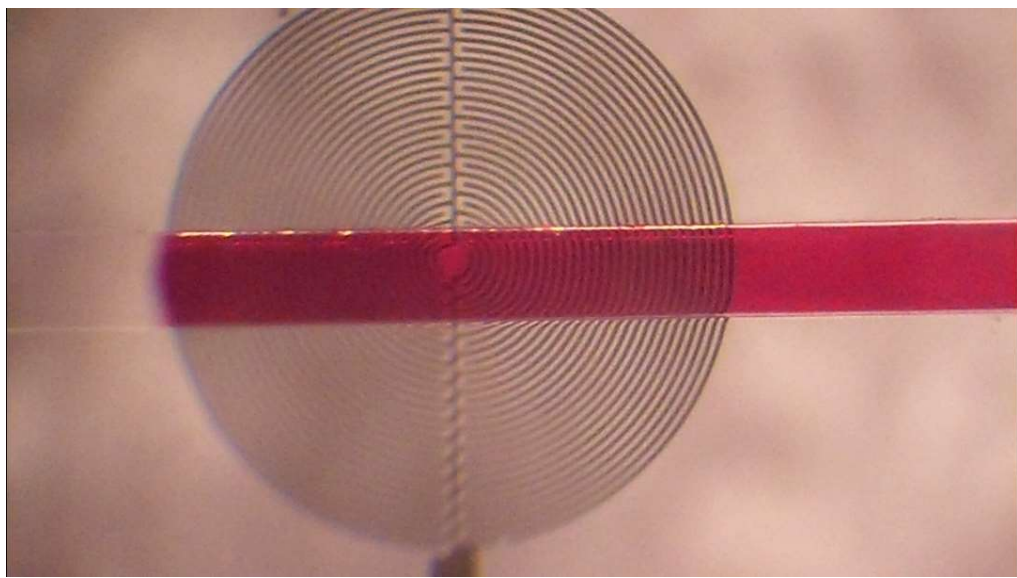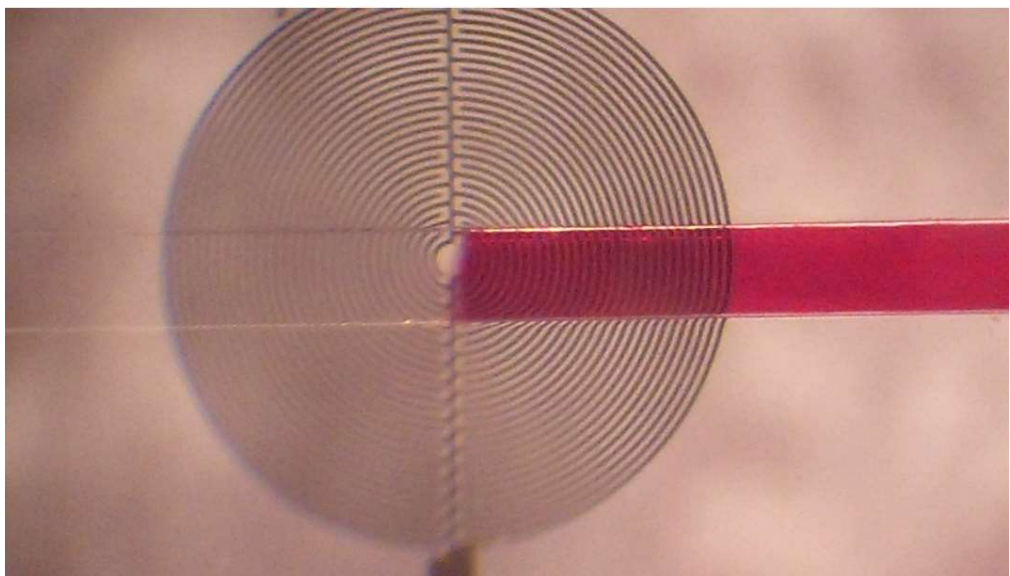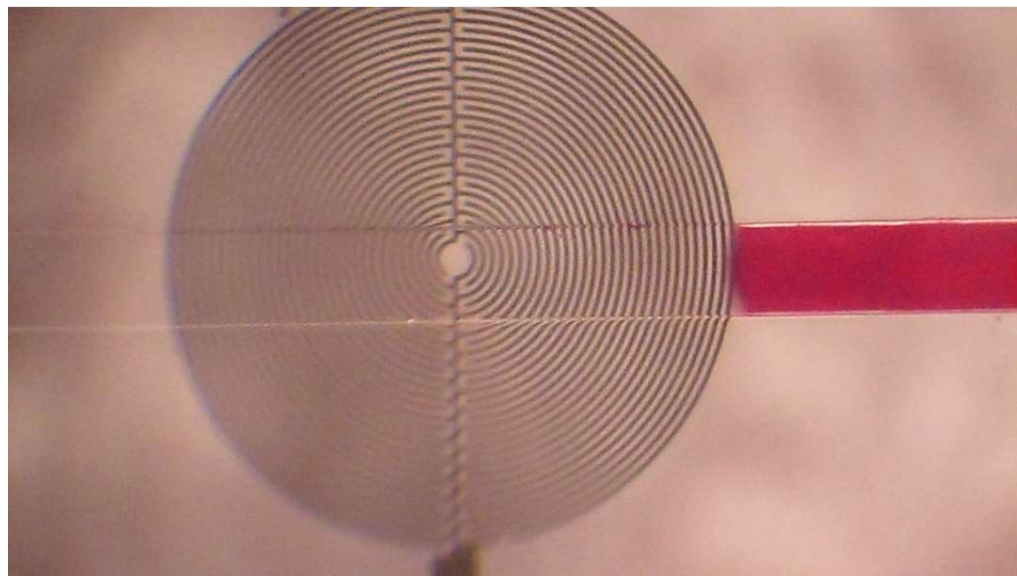

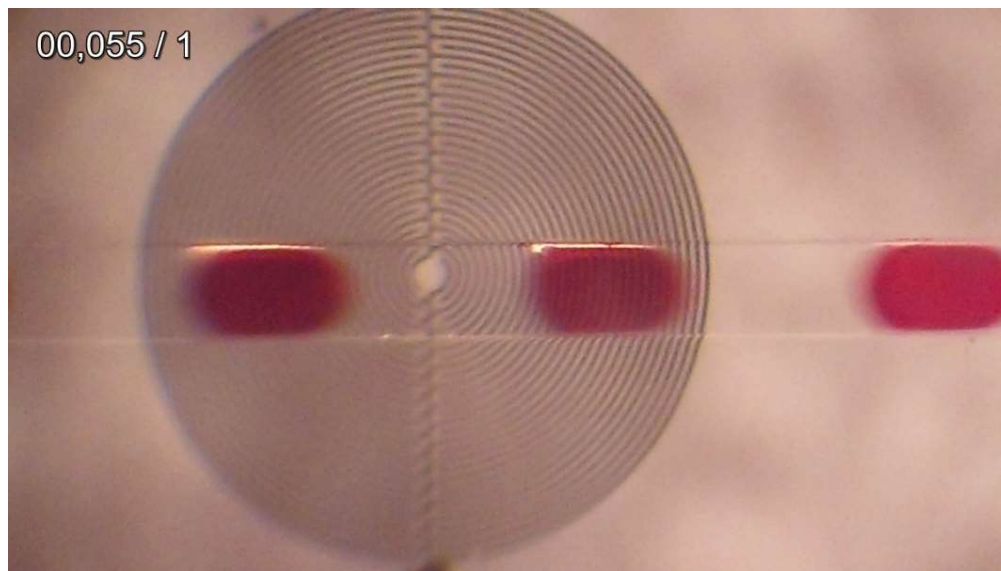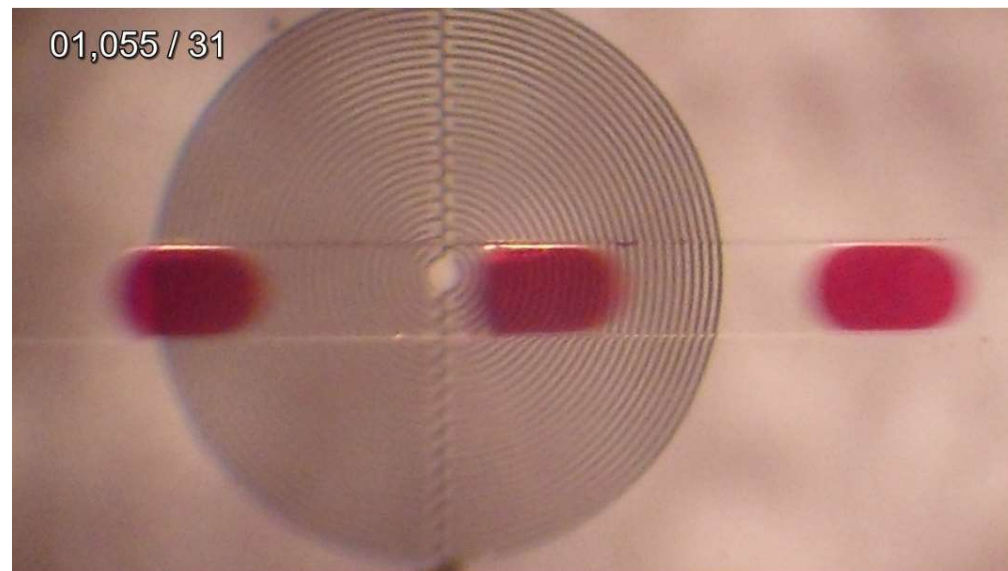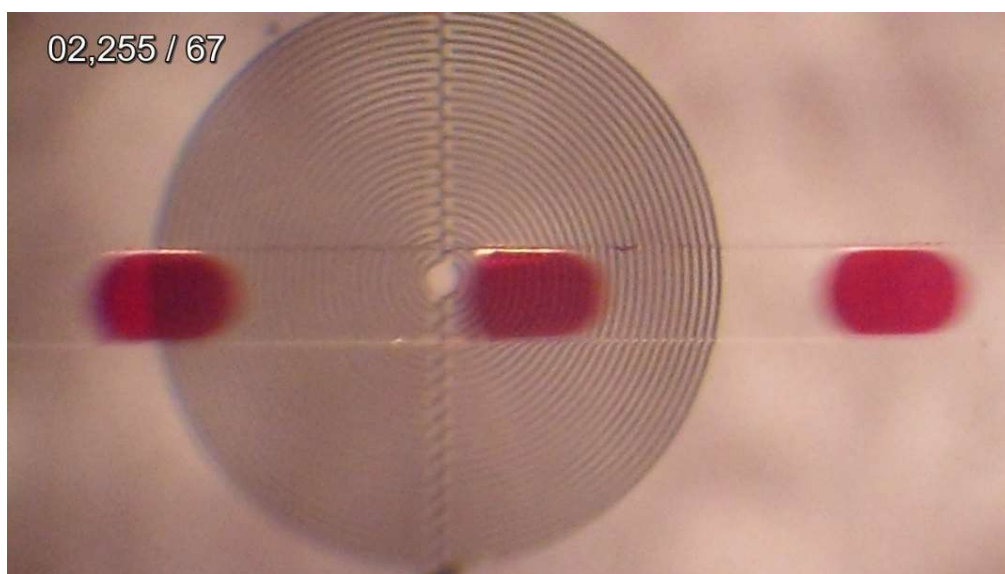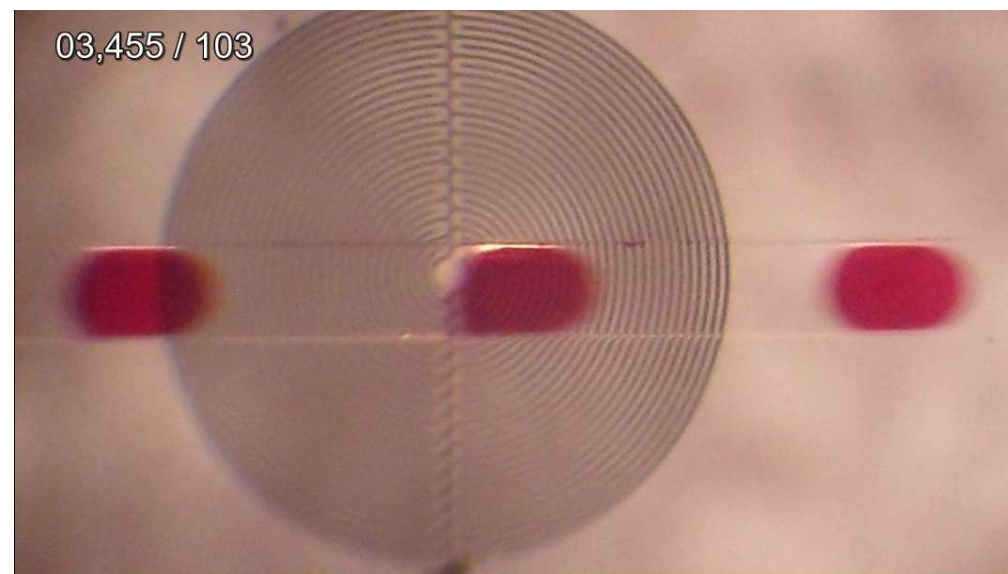

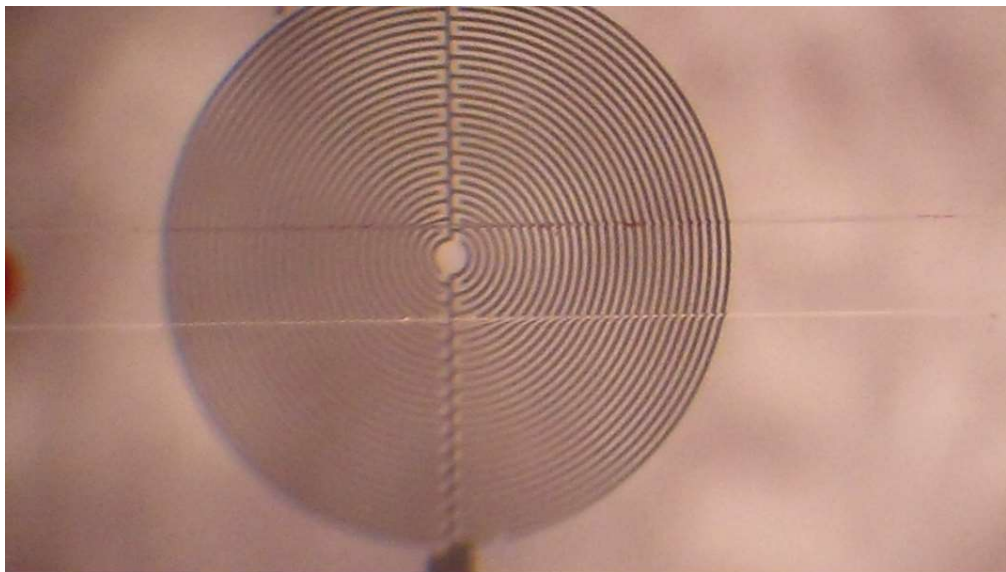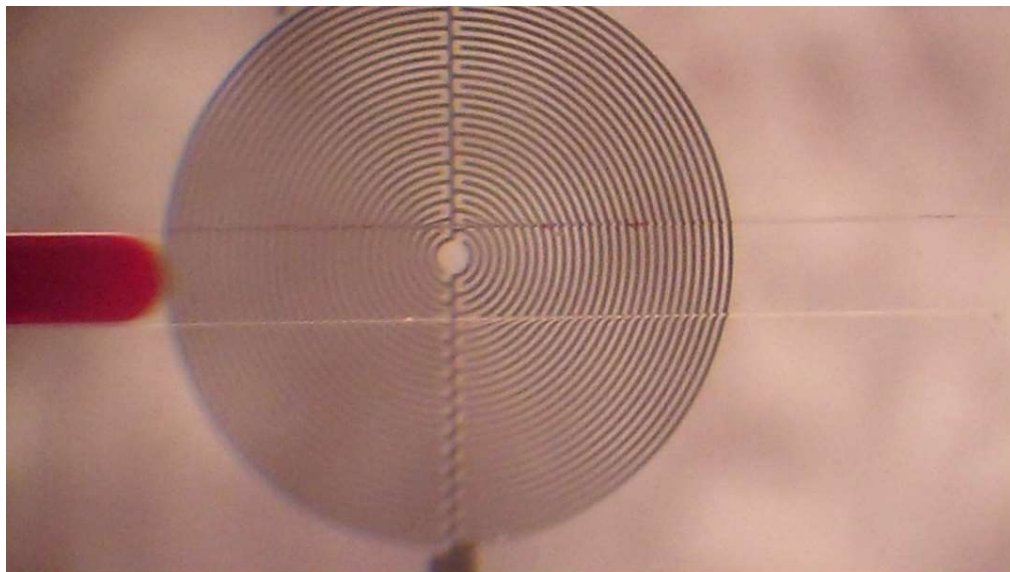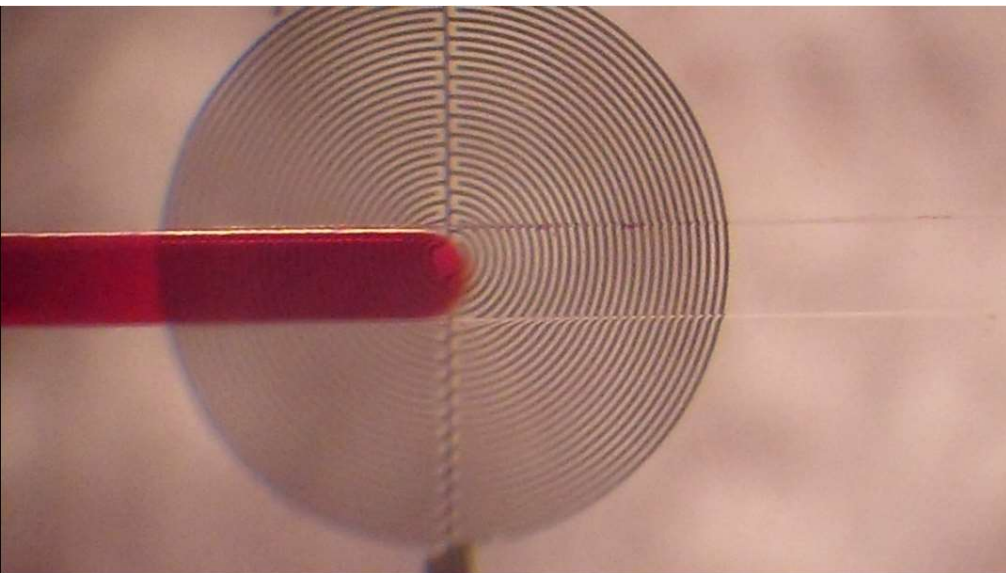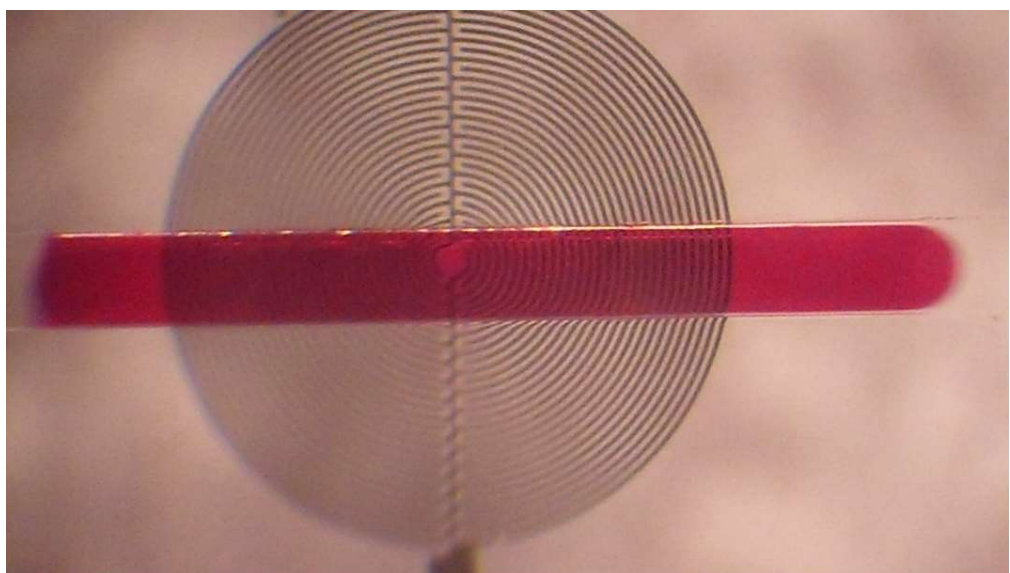

04,735 / 142

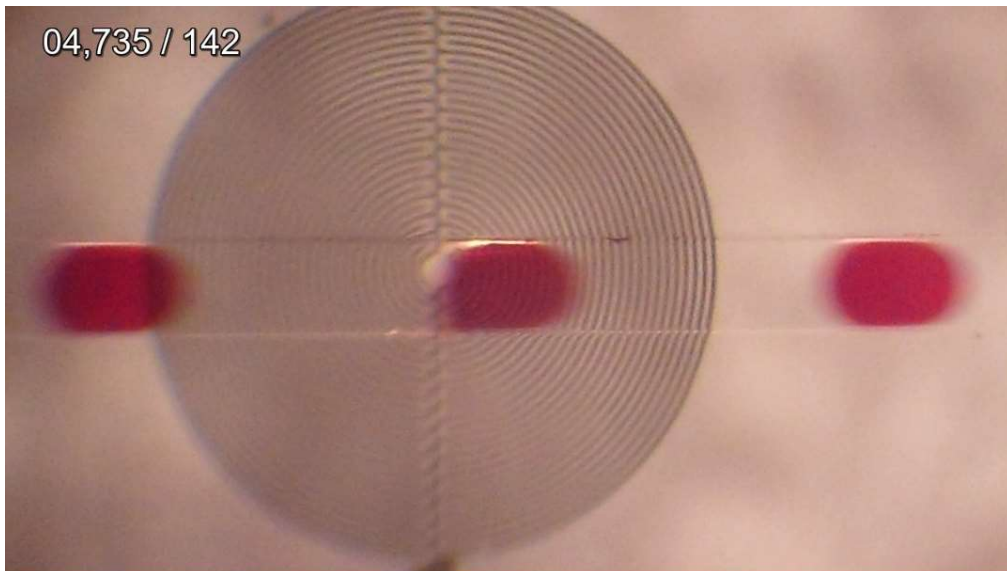

05,735 / 172

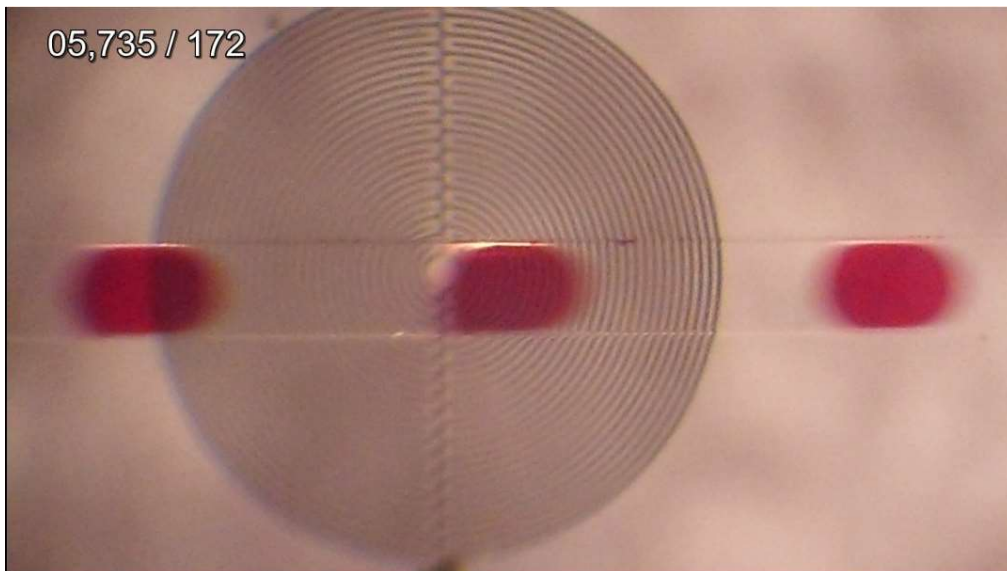

06,735 / 202

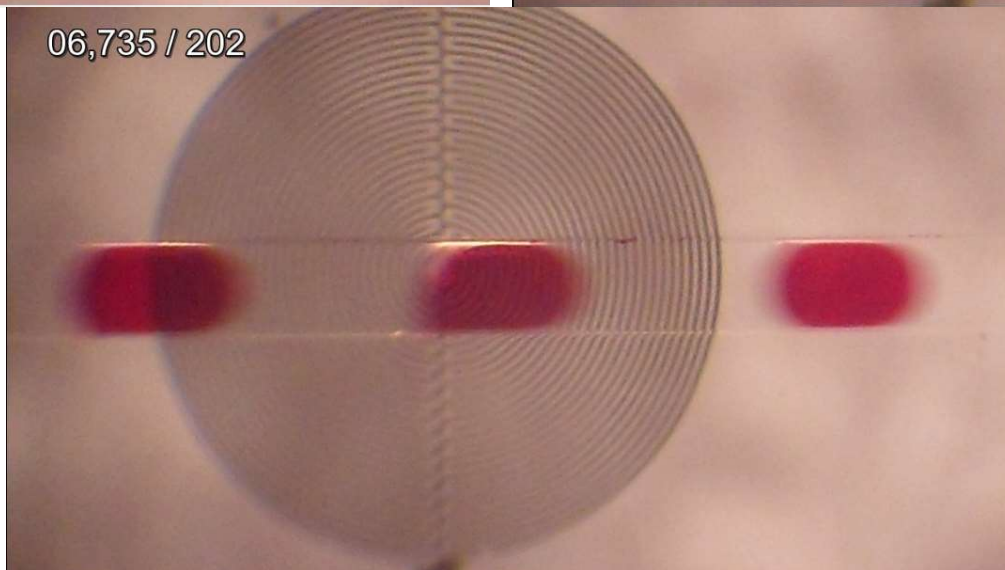

15,914 / 477

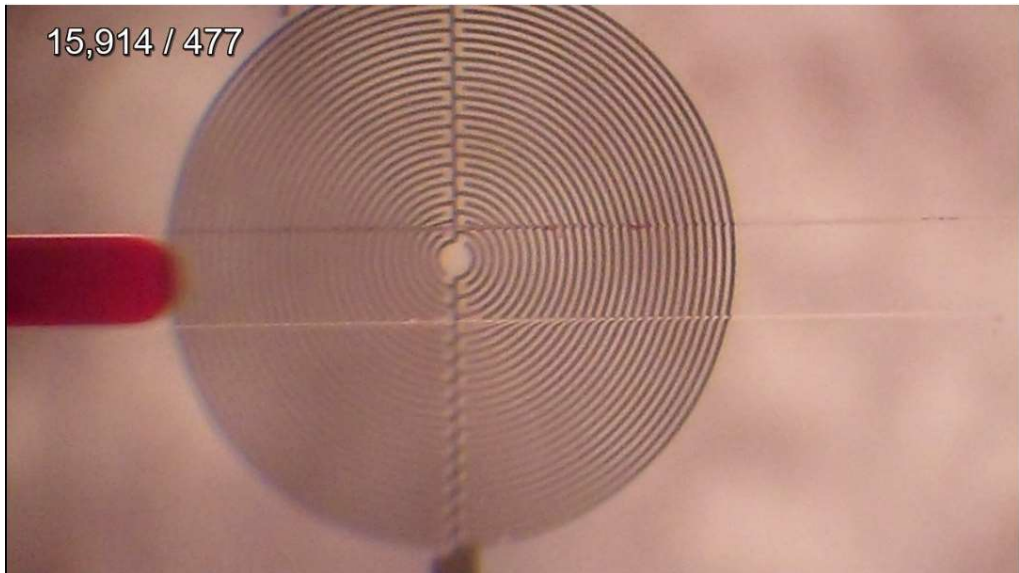

16,647 / 499

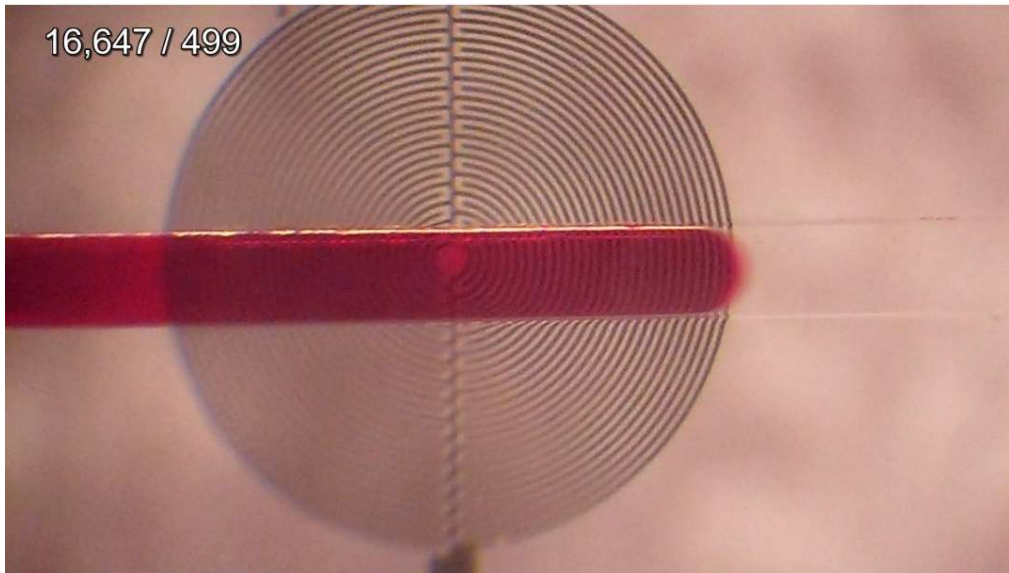

17,447 / 523

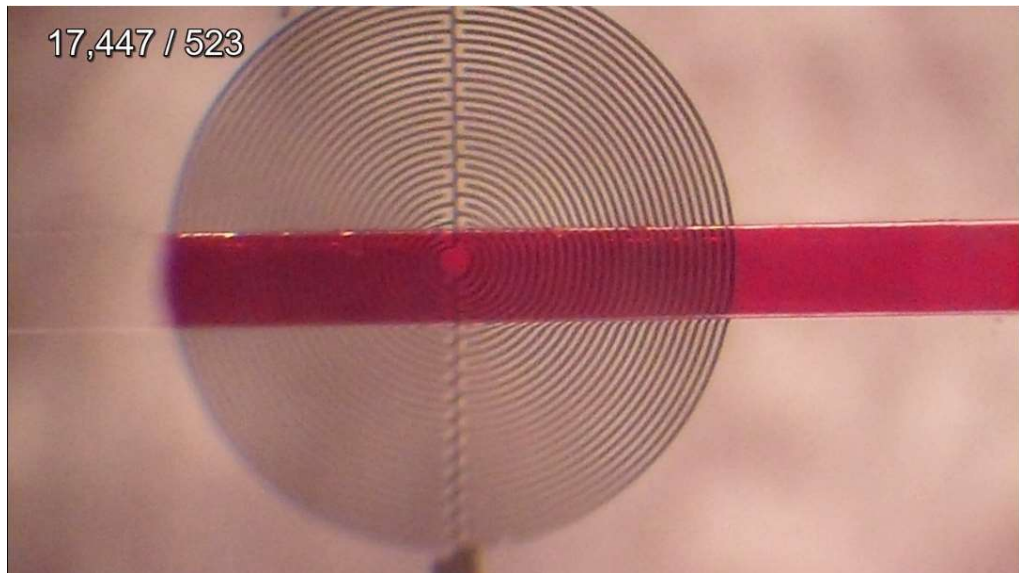

18,185 / 545

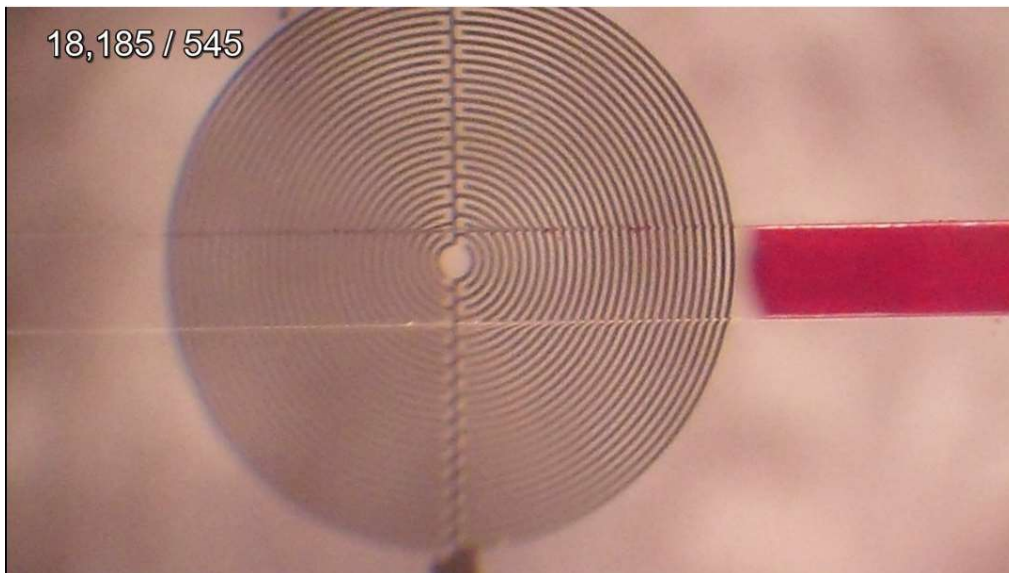

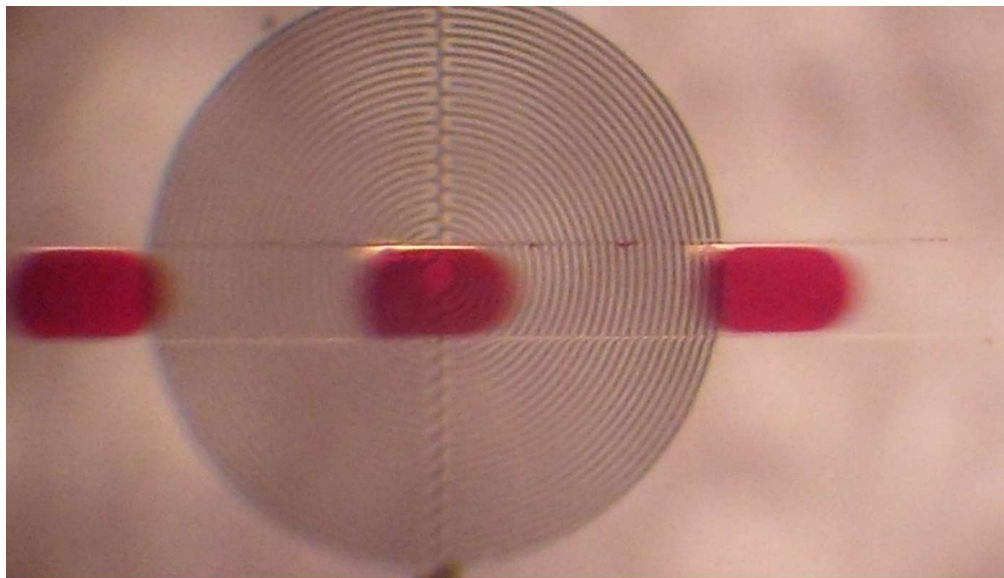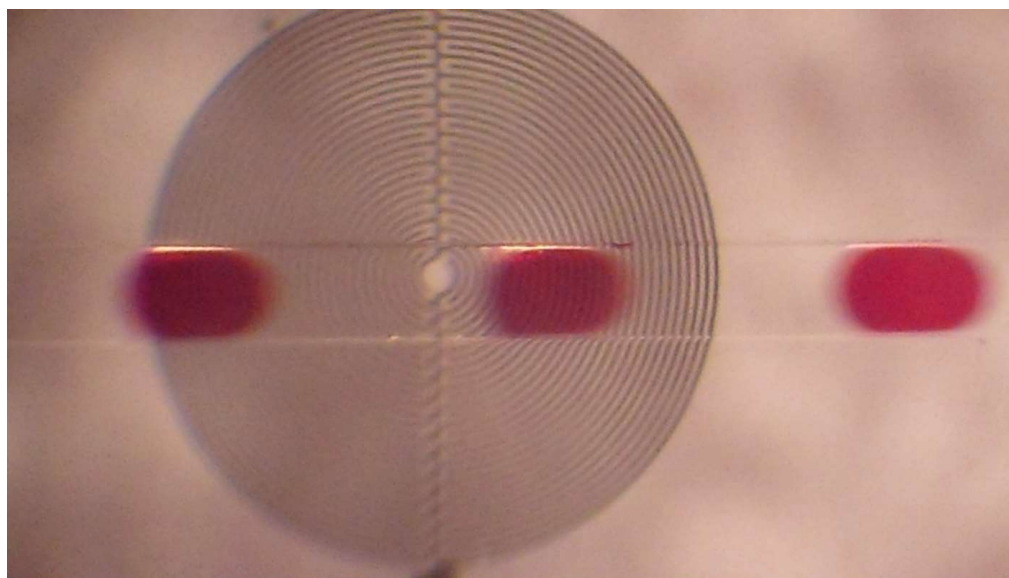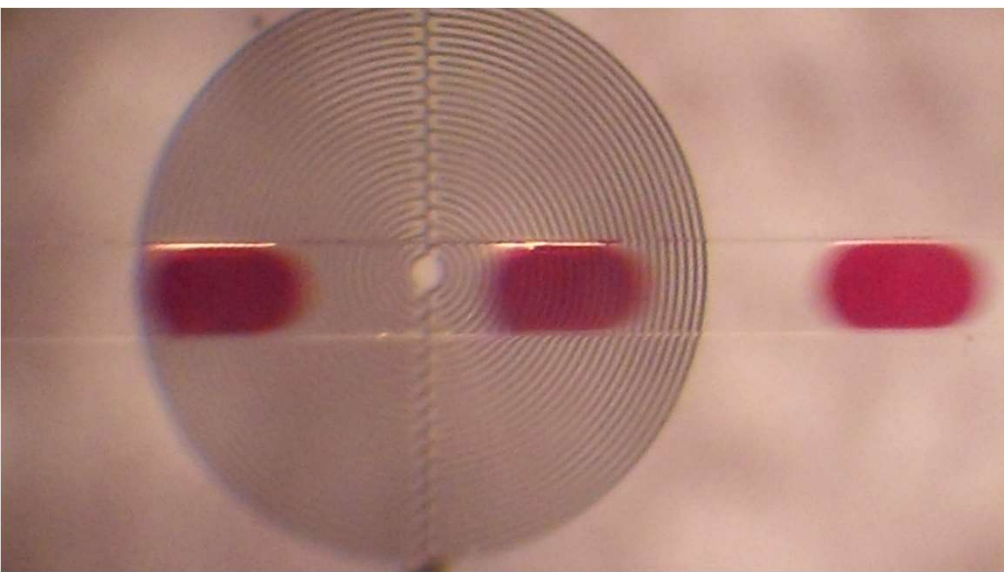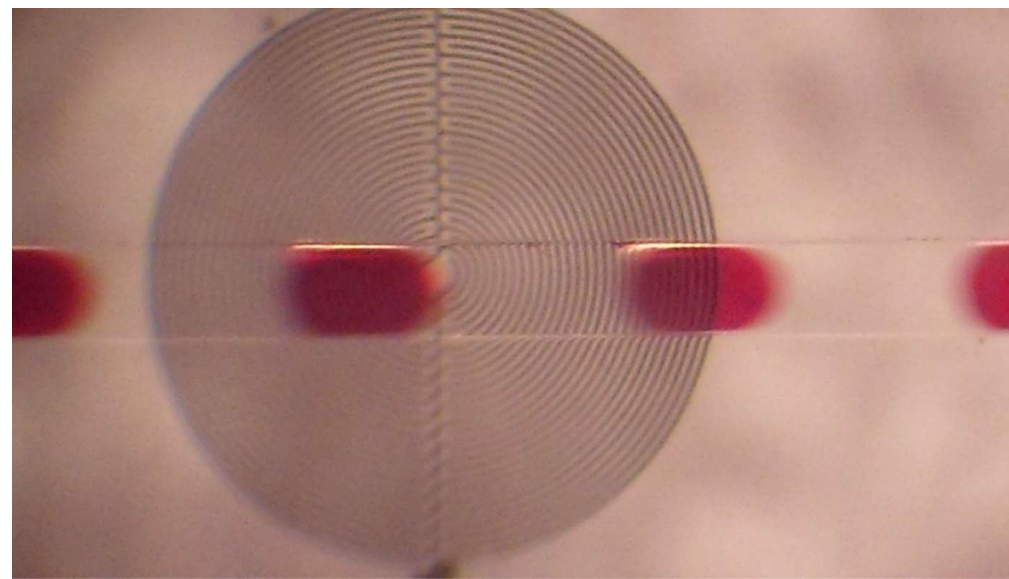

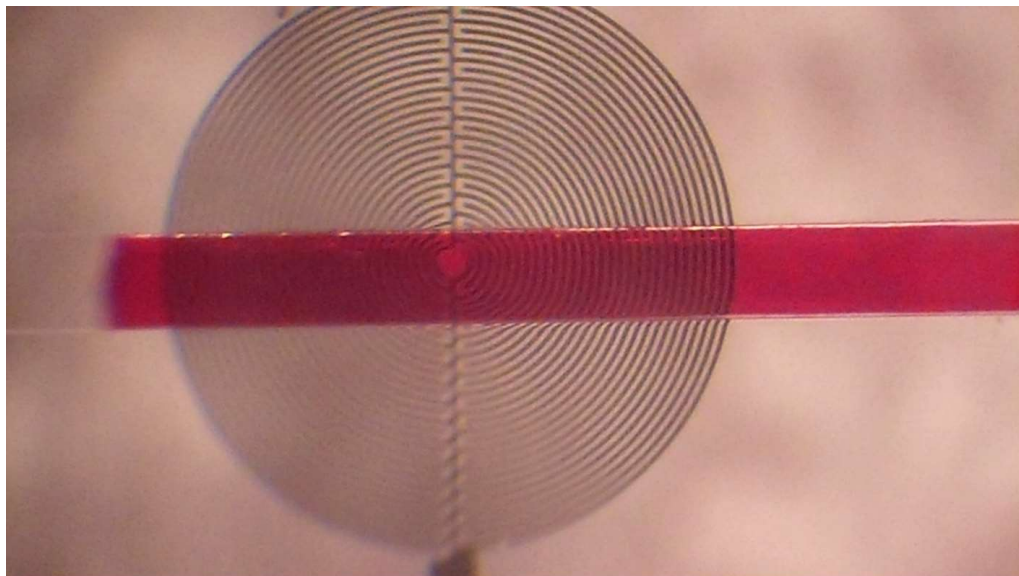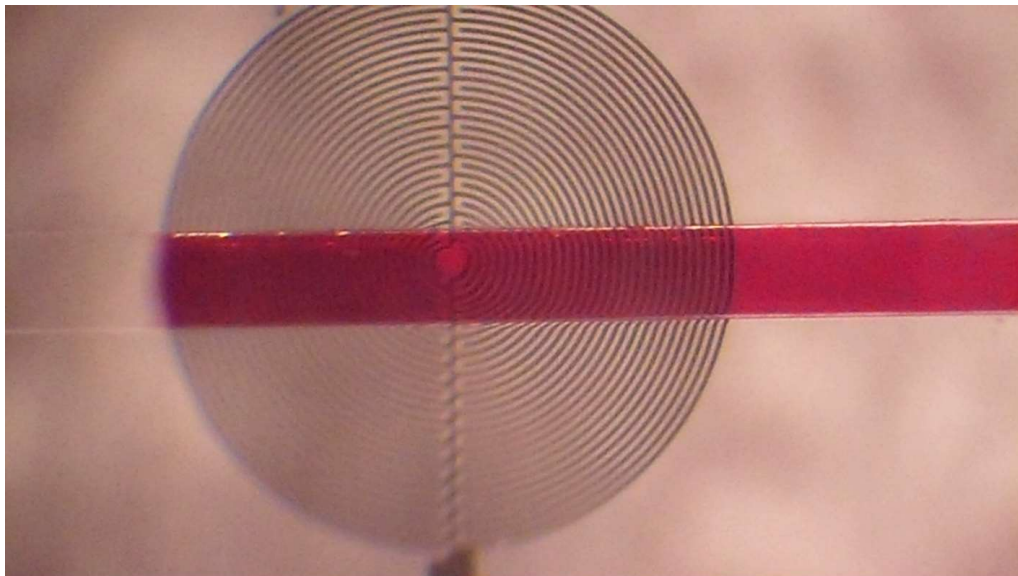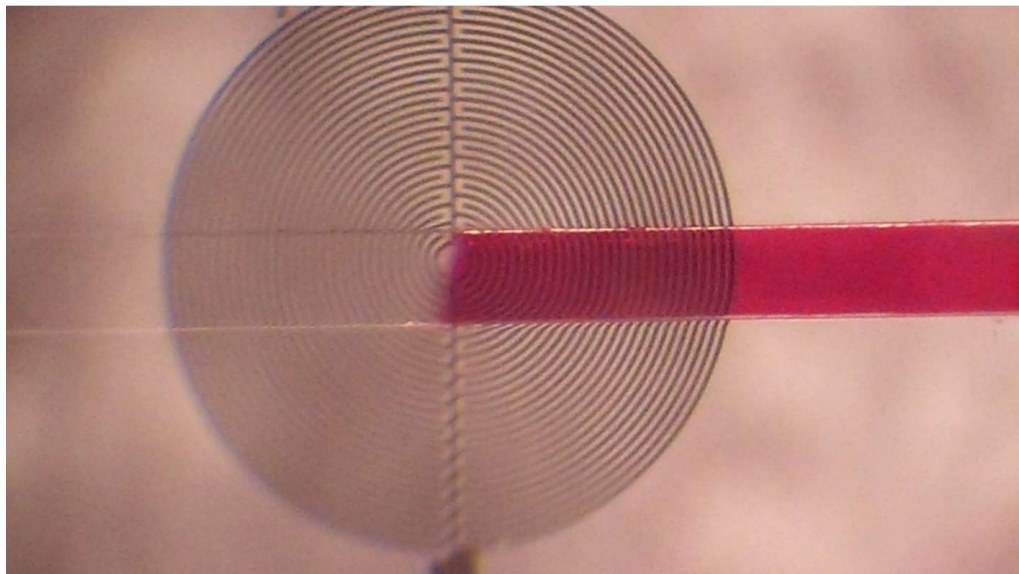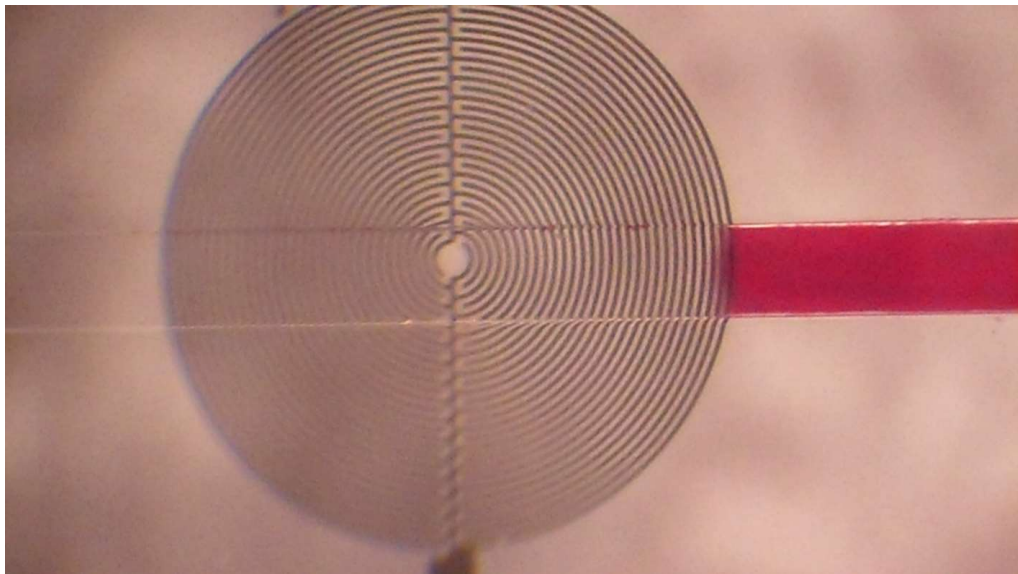

Supplement: Supplementary file 1 [file micromachines-15-00672-s001.zip › Supplementary File S1_droplets.pdf]
